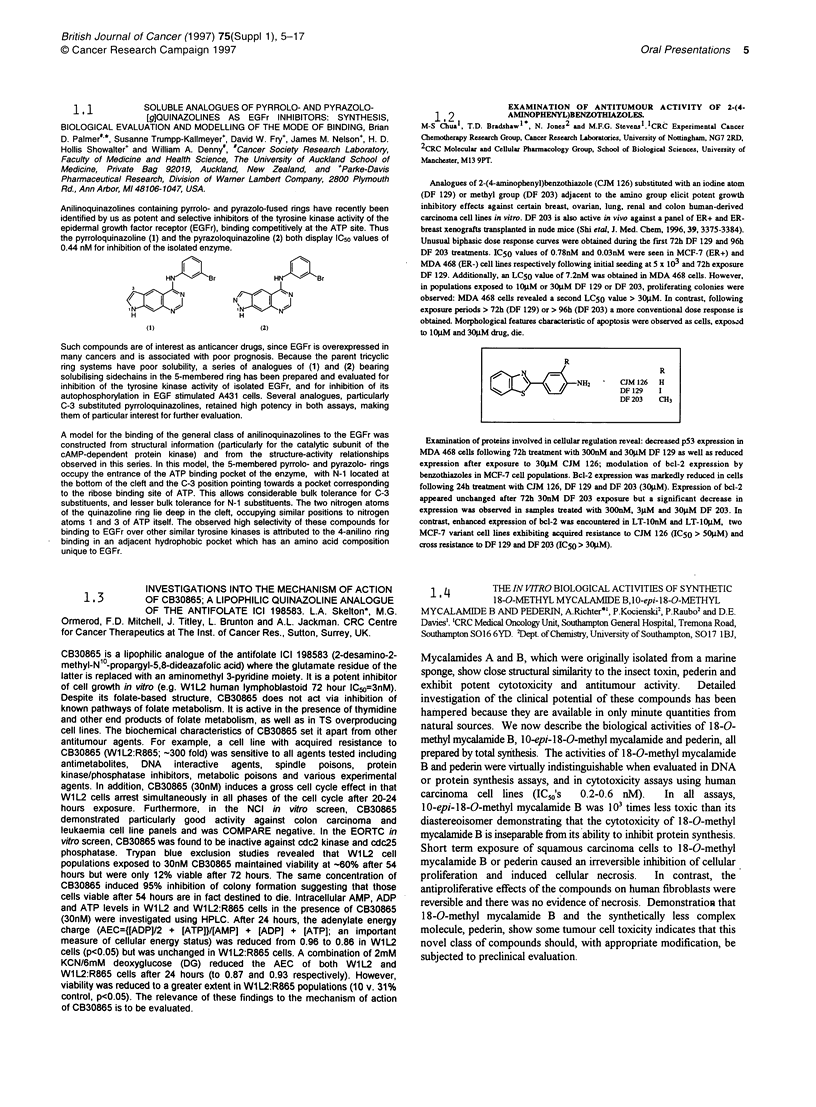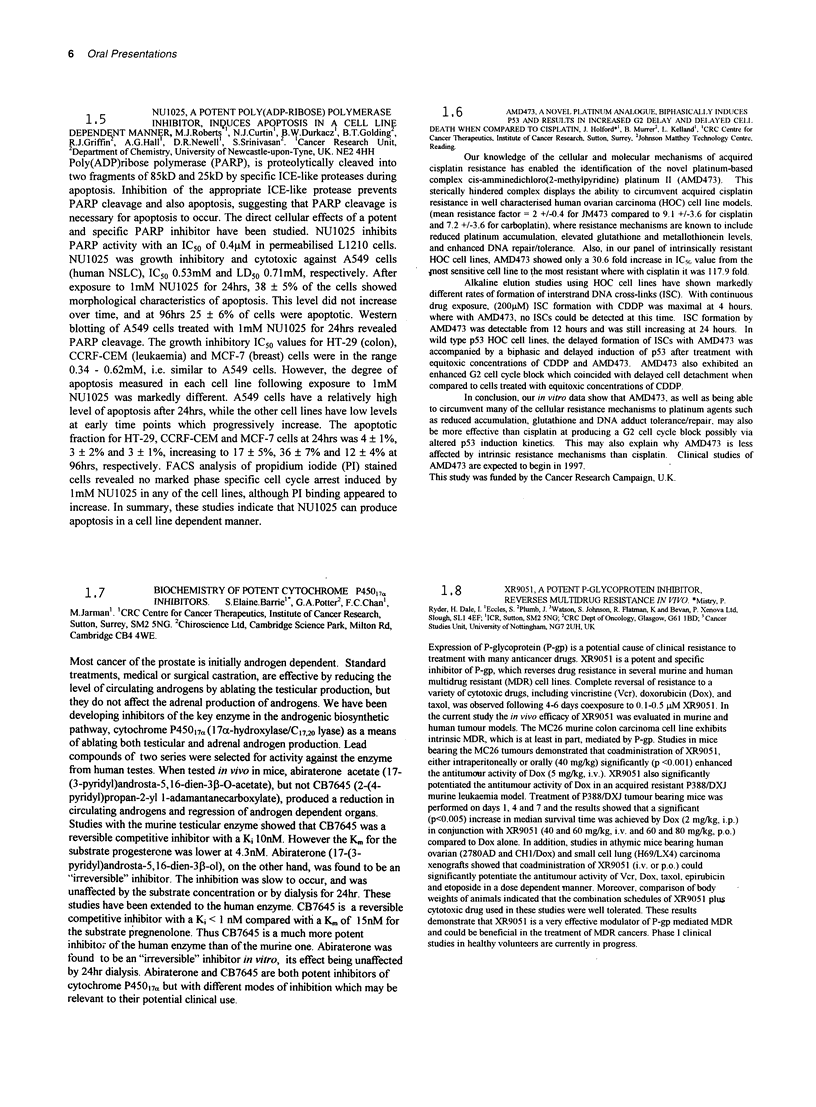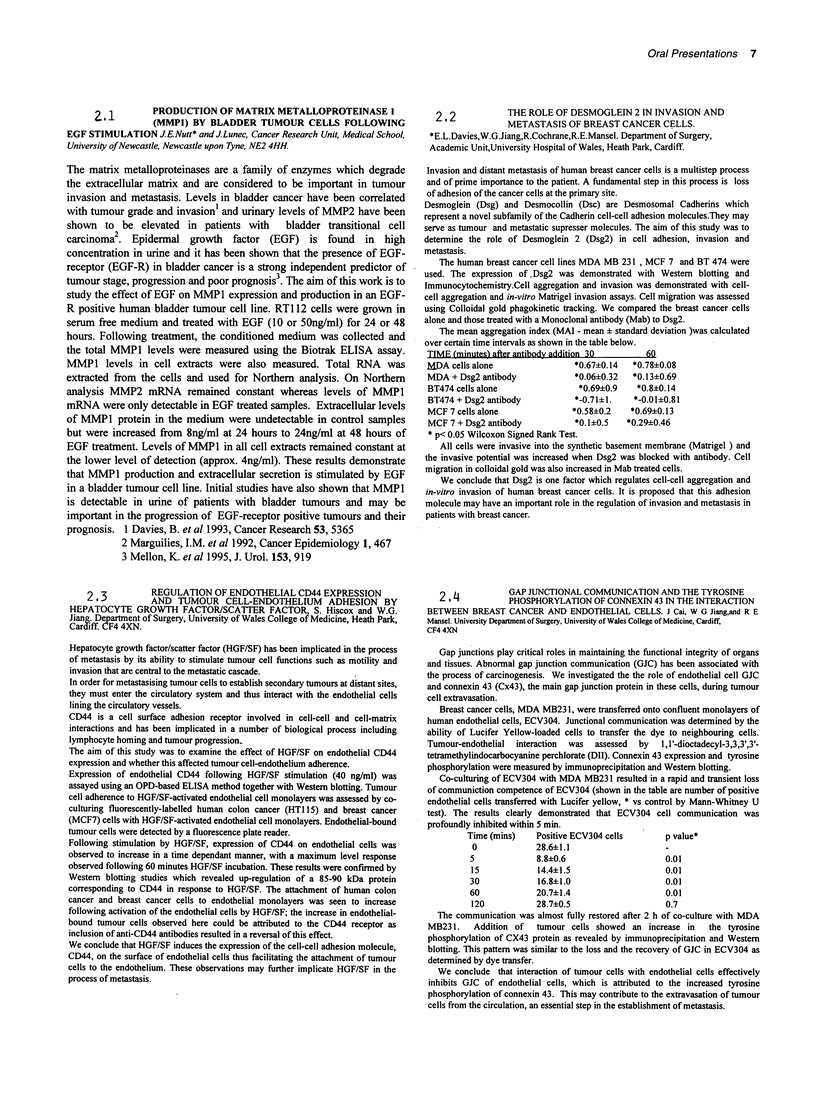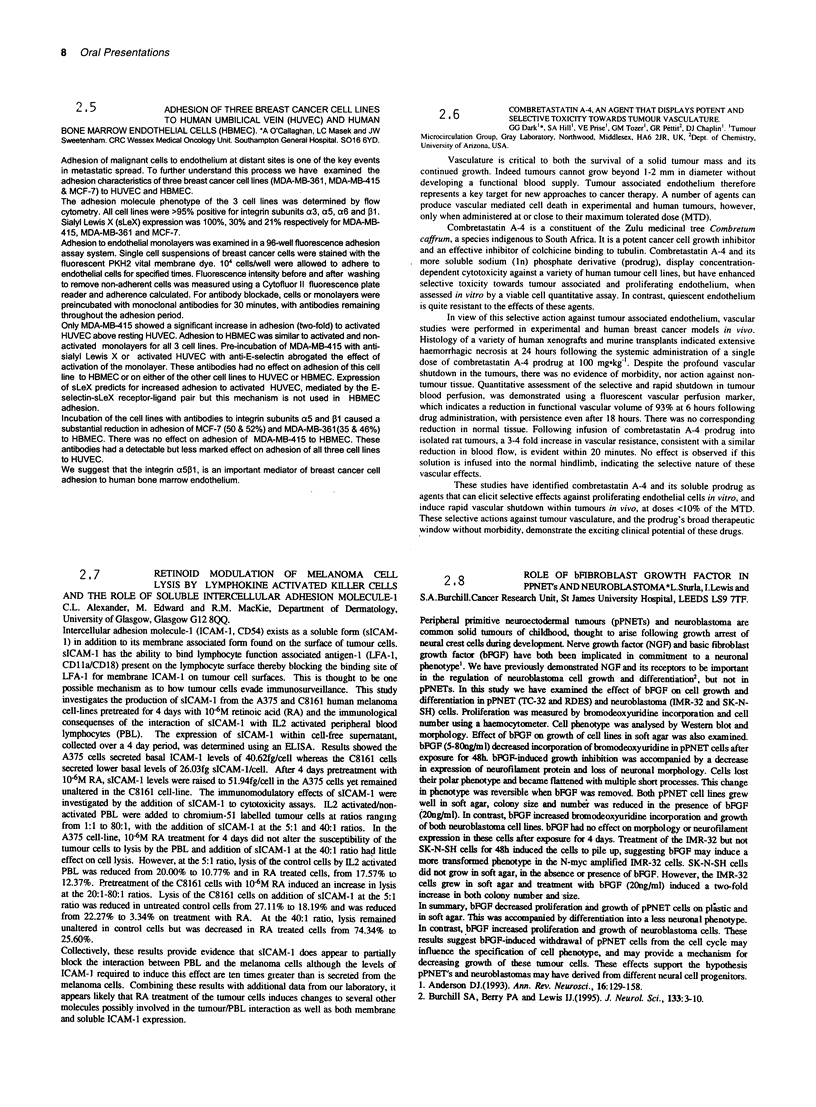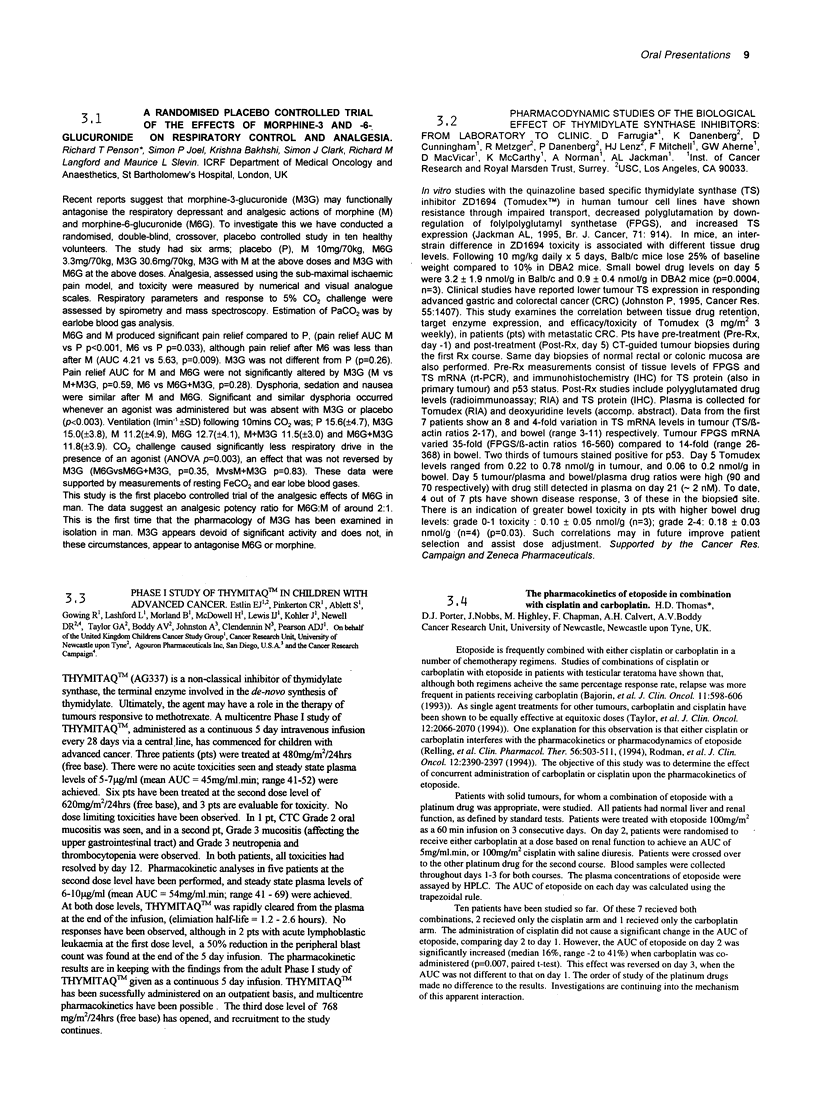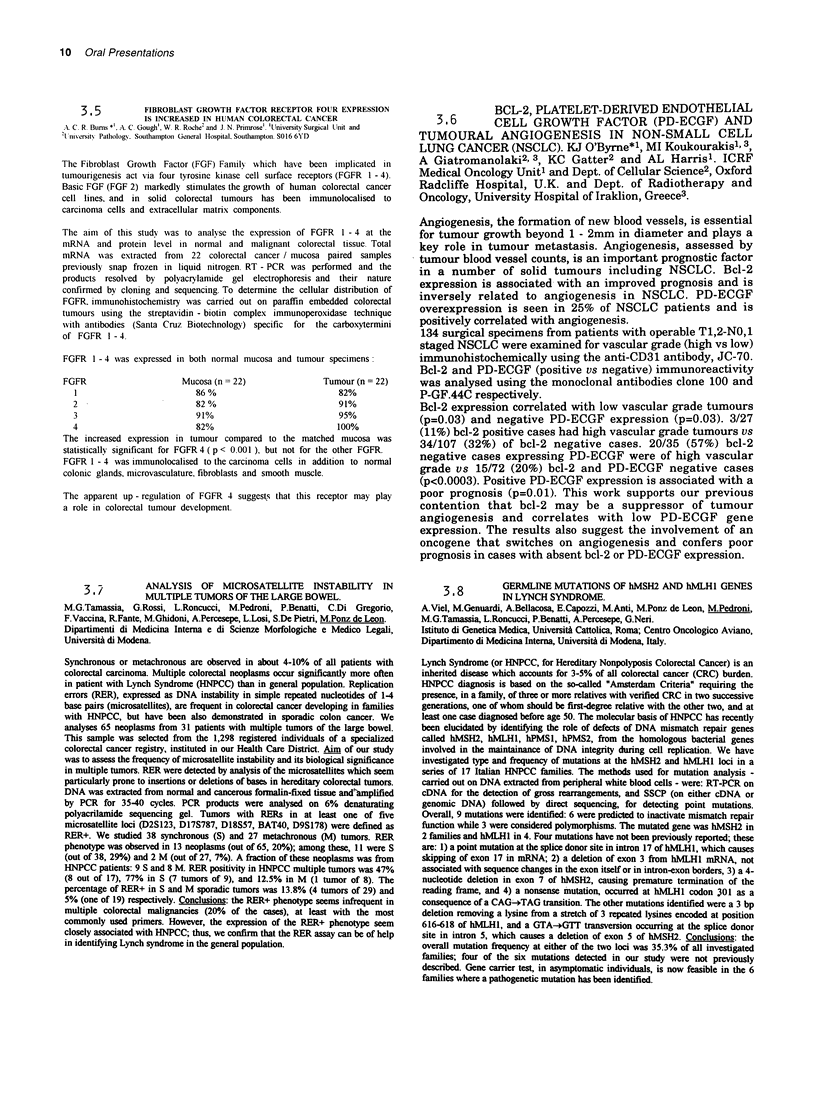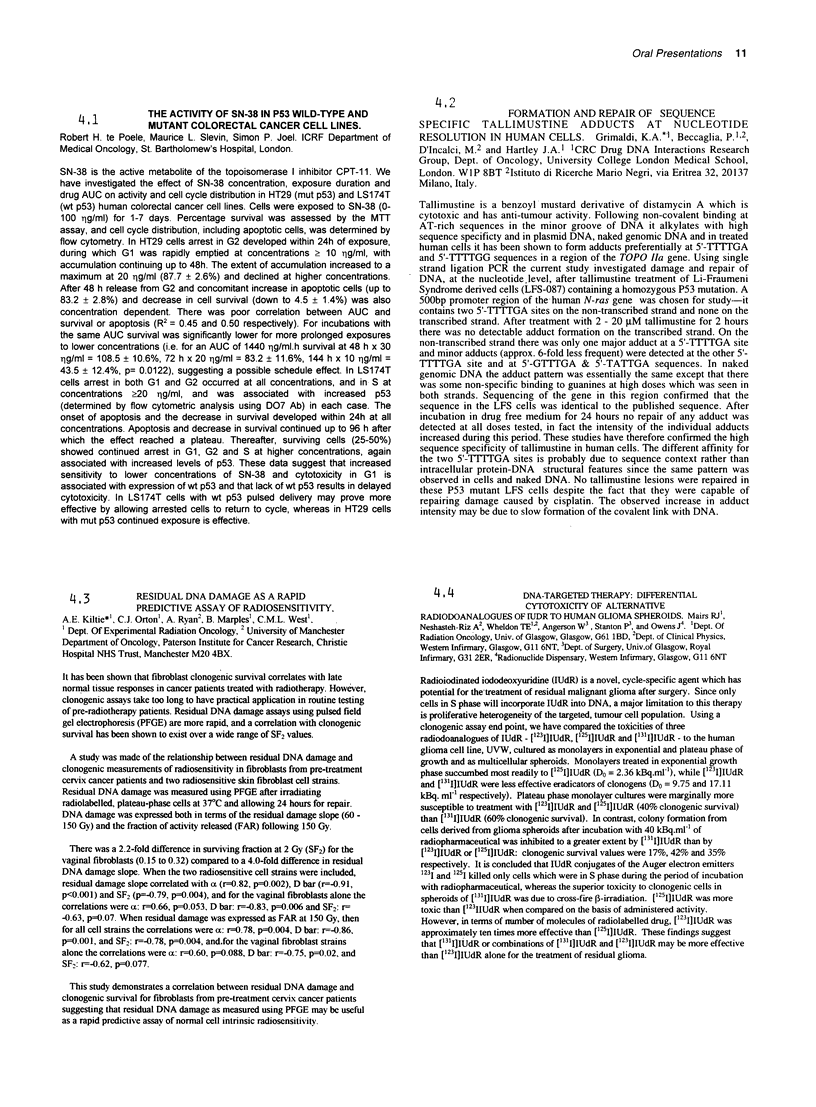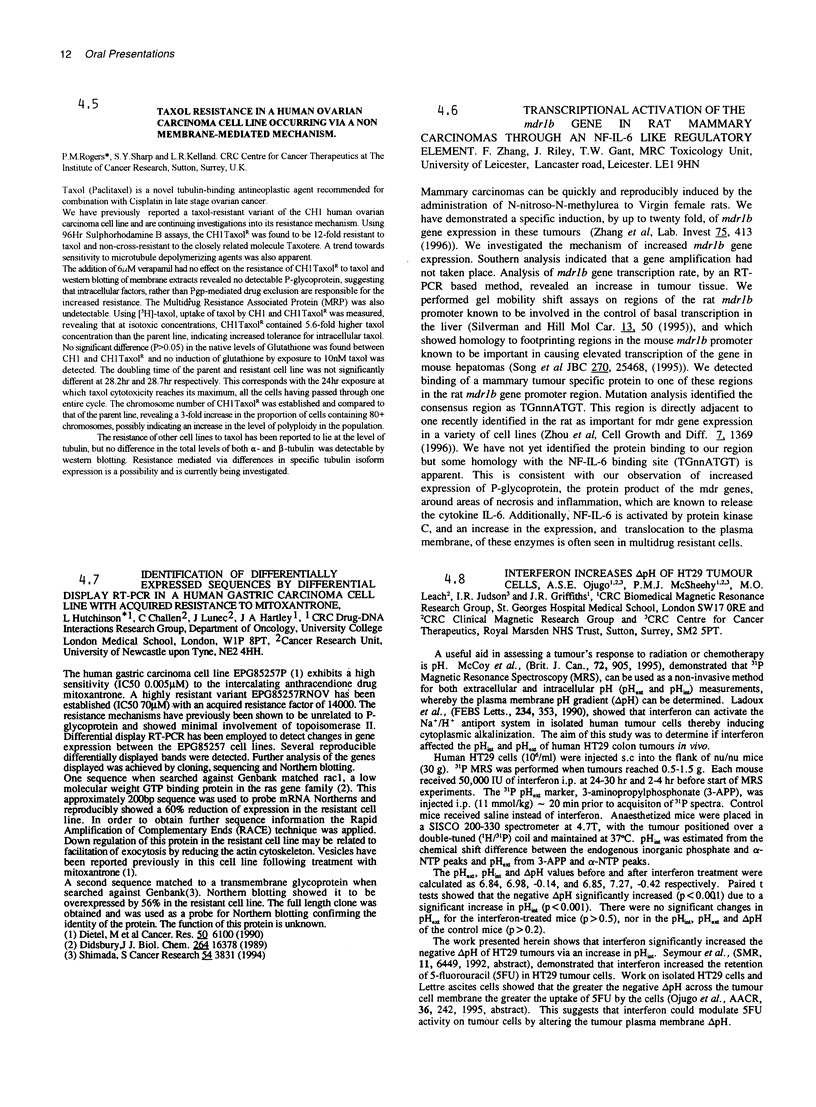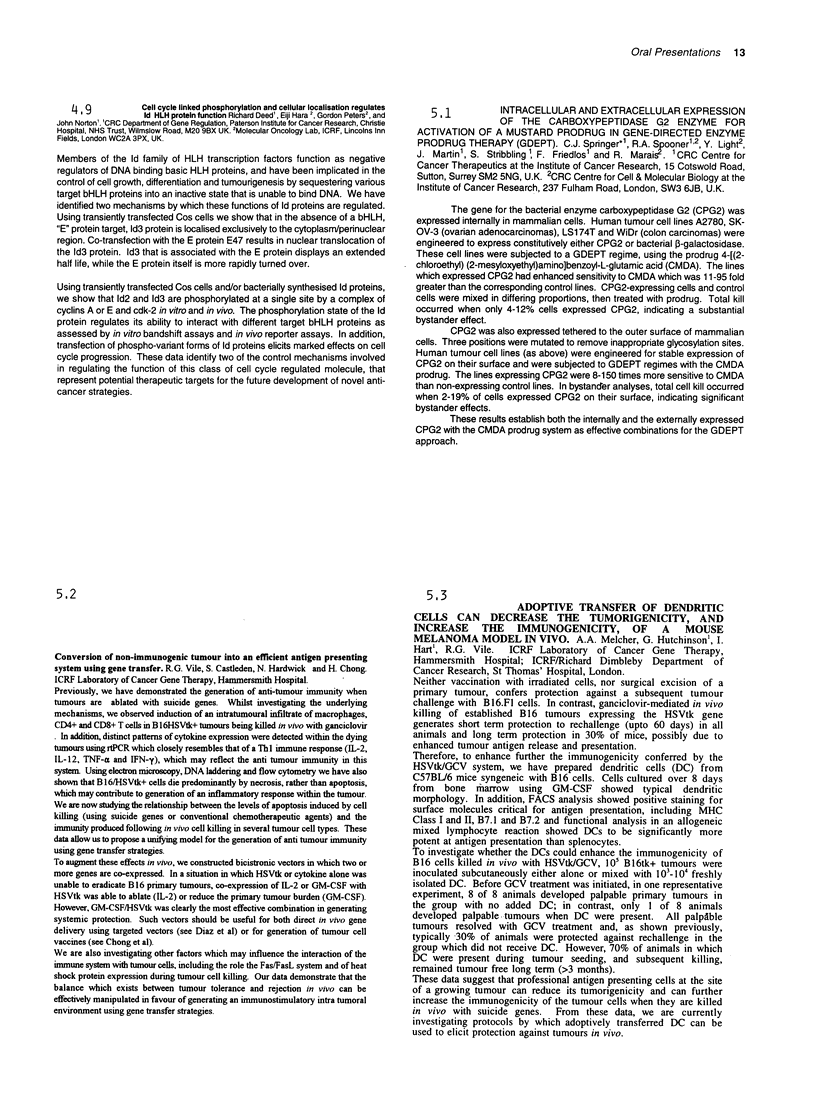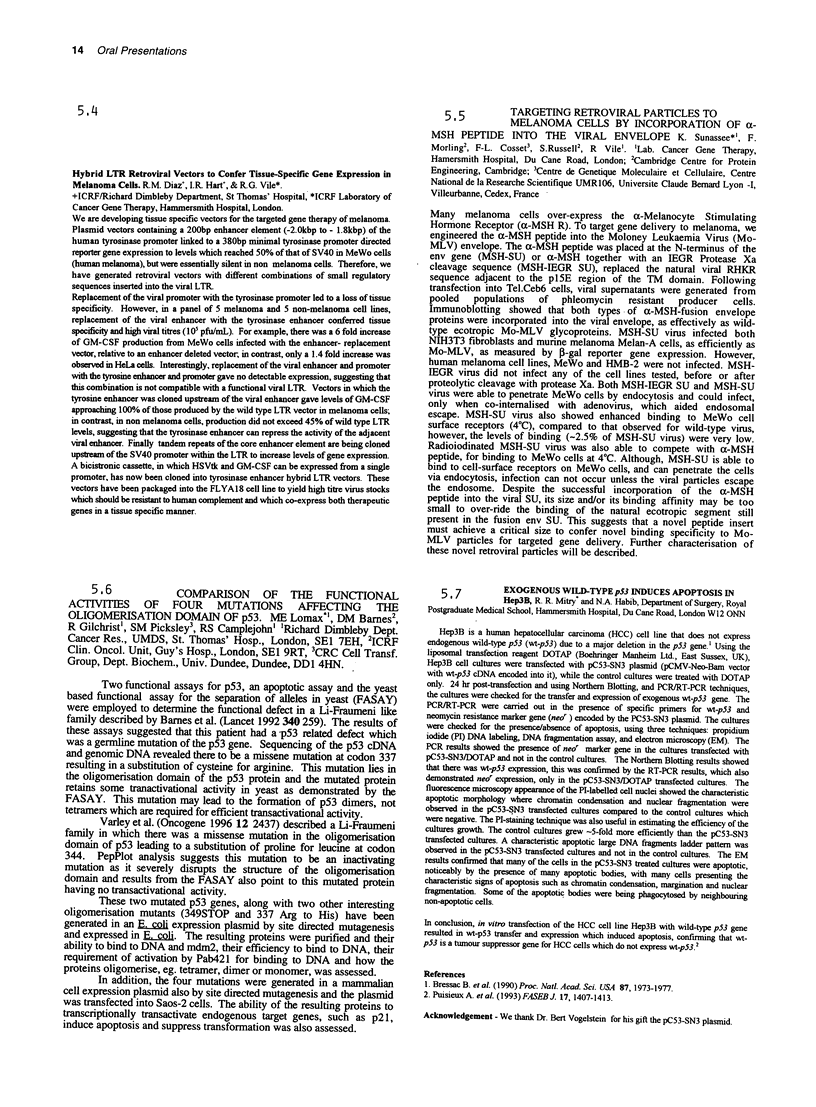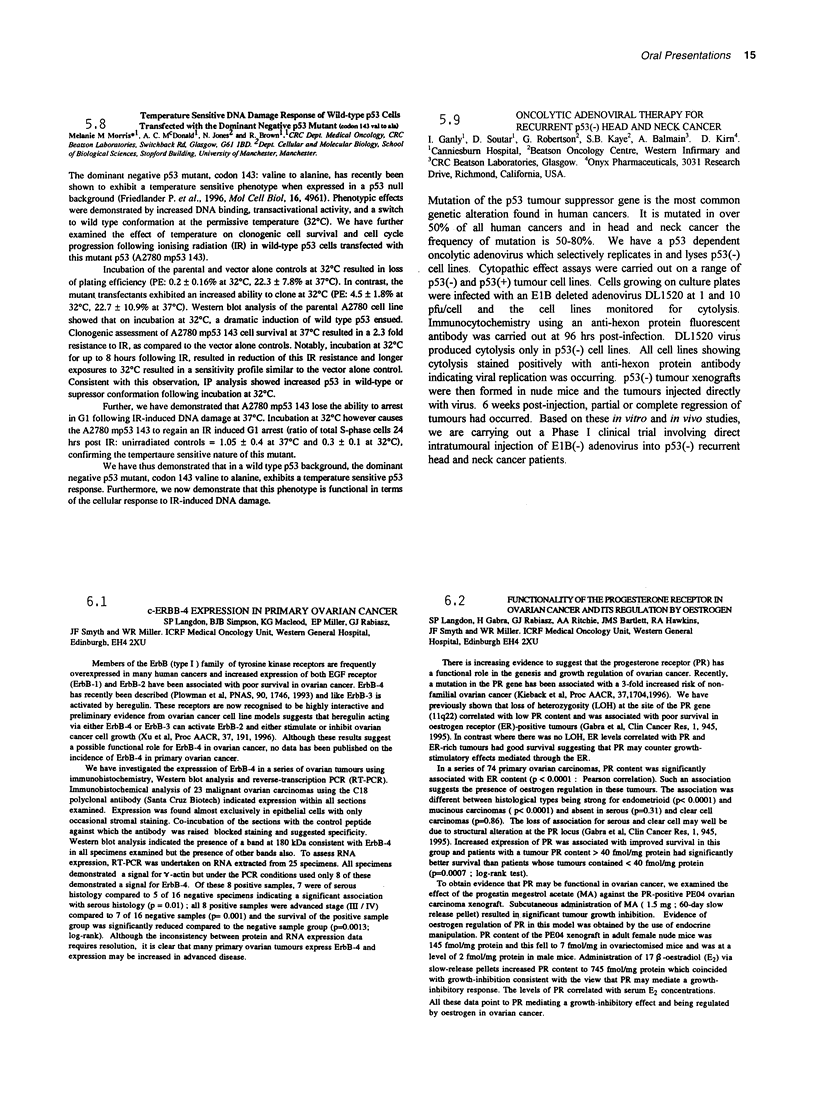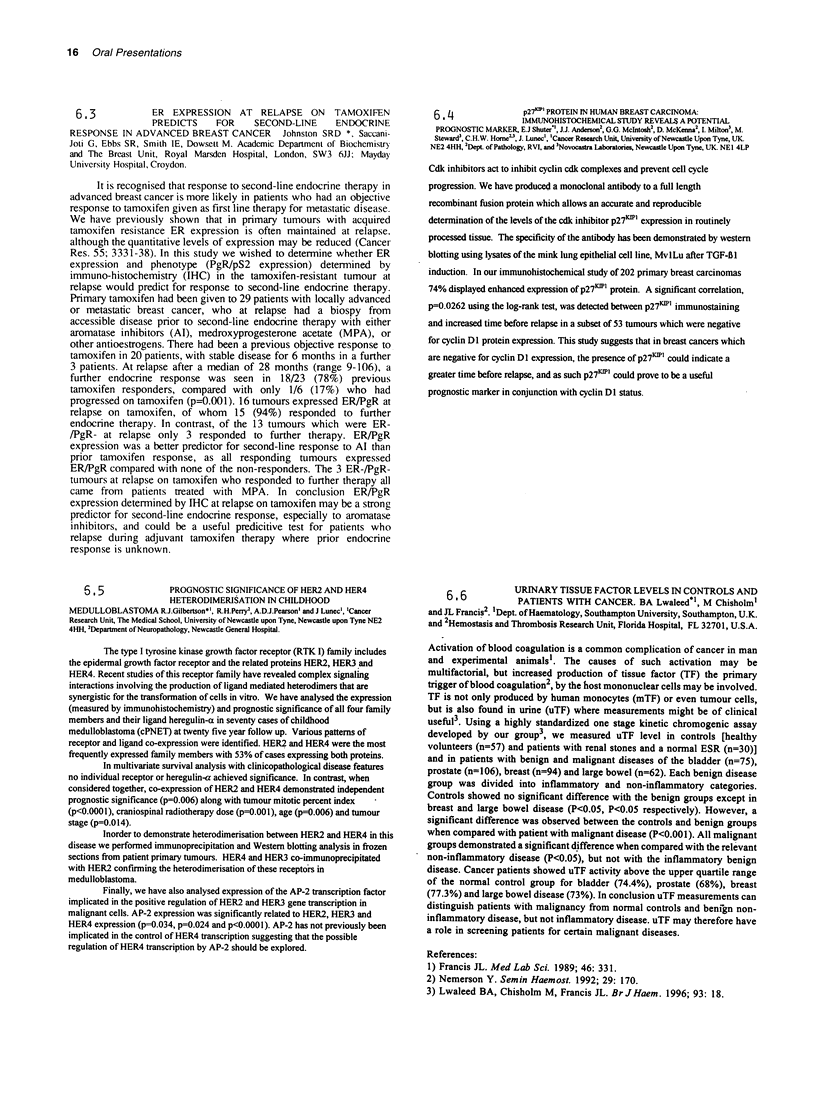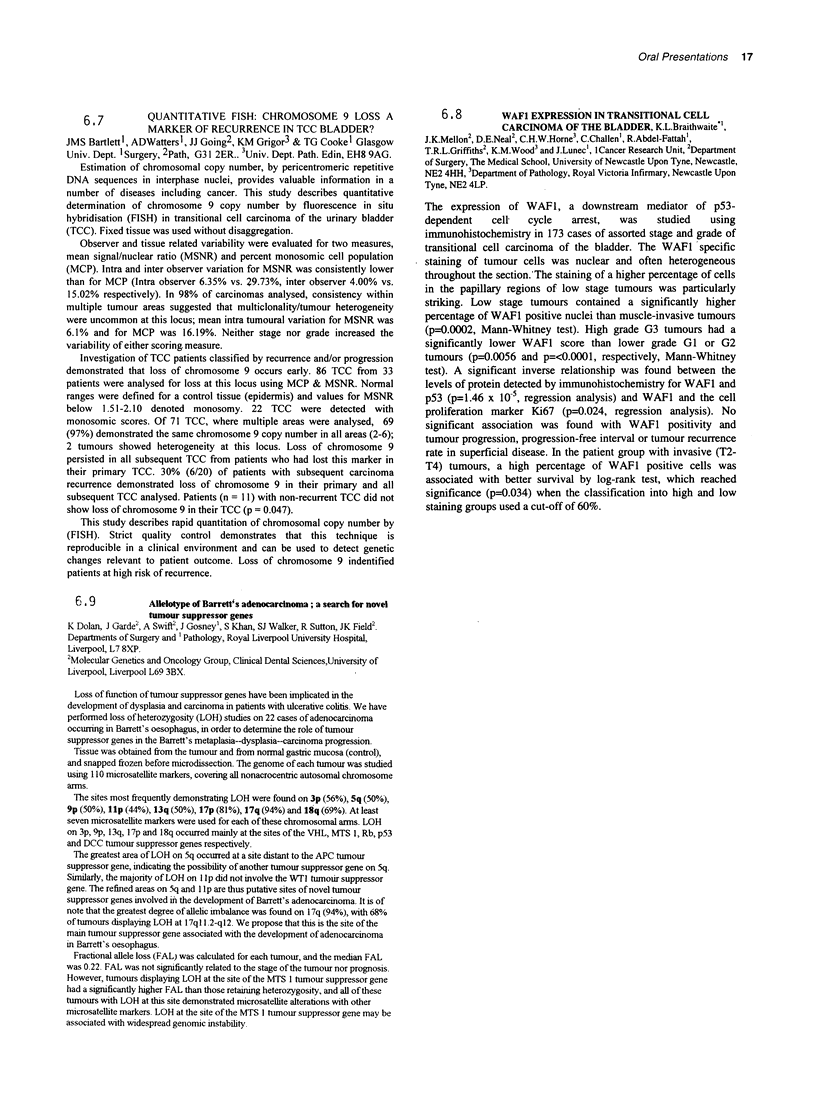# Oral presentations

**Published:** 1997

**Authors:** 


1.1 SOLUBLE ANALOGUES OF PYRROLO- AND PYRAZOLO-[*g*]QUINAZOLINES AS EGFr INHIBITORS: SYNTHESIS, BIOLOGICAL EVALUATION AND MODELLING OF THE MODE OF BINDING,

Brian D. Palmer[#,*], Susanne Trumpp-Kallmeyer[+], David W. Fry[+], James M. Nelson[+], H. D. Hollis Showalter[+] and William A. Denny[#], [#]*Cancer Society Research Laboratory, Faculty of Medicine and Health Science, The University of Auckland School of Medicine, Private Bag 92019, Auckland, New Zealand, and* [+]*Parke-Davis Pharmaceutical Research, Division of Warner Lambert Company, 2800 Plymouth Rd., Ann Arbor, MI 48106-1047, USA.*

Anilinoquinazolines containing pyrrolo- and pyrazolo-fused rings have recently been identified by us as potent and selective inhibitors of the tyrosine kinase activity of the epidermal growth factor receptor (EGFr), binding competitively at the ATP site. Thus the pyrroloquinazoline (1) and the pyrazoloquinazoline (2) both display $IC_{50}$ values of 0.44 nM for inhibition of the isolated enzyme.

(1)

(2)

Such compounds are of interest as anticancer drugs, since EGFr is overexpressed in many cancers and is associated with poor prognosis. Because the parent tricyclic ring systems have poor solubility, a series of analogues of (1) and (2) bearing solubilising sidechains in the 5-membered ring has been prepared and evaluated for inhibition of the tyrosine kinase activity of isolated EGFr, and for inhibition of its autophosphorylation in EGF stimulated A431 cells. Several analogues, particularly C-3 substituted pyrroloquinazolines, retained high potency in both assays, making them of particular interest for further evaluation.

A model for the binding of the general class of anilinoquinazolines to the EGFr was constructed from structural information (particularly for the catalytic subunit of the cAMP-dependent protein kinase) and from the structure-activity relationships observed in this series. In this model, the 5-membered pyrrolo- and pyrazolo- rings occupy the entrance of the ATP binding pocket of the enzyme, with N-1 located at the bottom of the cleft and the C-3 position pointing towards a pocket corresponding to the ribose binding site of ATP. This allows considerable bulk tolerance for C-3 substituents, and lesser bulk tolerance for N-1 substituents. The two nitrogen atoms of the quinazoline ring lie deep in the cleft, occupying similar positions to nitrogen atoms 1 and 3 of ATP itself. The observed high selectivity of these compounds for binding to EGFr over other similar tyrosine kinases is attributed to the 4-anilino ring binding in an adjacent hydrophobic pocket which has an amino acid composition unique to EGFr.

1.2 **EXAMINATION OF ANTITUMOUR ACTIVITY OF 2-(4-AMINOPHENYL)BENZOTHIAZOLES.**

**M-S Chua[1], T.D. Bradshaw[1*], N. Jones[2] and M.F.G. Stevens[1]. [1]CRC Experimental Cancer Chemotherapy Research Group, Cancer Research Laboratories, University of Nottingham, NG7 2RD, [2]CRC Molecular and Cellular Pharmacology Group, School of Biological Sciences, University of Manchester, M13 9PT.**

**Analogues of 2-(4-aminophenyl)benzothiazole (CJM 126) substituted with an iodine atom (DF 129) or methyl group (DF 203) adjacent to the amino group elicit potent growth inhibitory effects against certain breast, ovarian, lung, renal and colon human-derived carcinoma cell lines *in vitro*. DF 203 is also active *in vivo* against a panel of ER+ and ER-breast xenografts transplanted in nude mice (Shi *etal*, J. Med. Chem, 1996, 39, 3375-3384). Unusual biphasic dose response curves were obtained during the first 72h DF 129 and 96h DF 203 treatments. $IC_{50}$ values of 0.78nM and 0.03nM were seen in MCF-7 (ER+) and MDA 468 (ER-) cell lines respectively following initial seeding at 5 x $10^3$ and 72h exposure DF 129. Additionally, an $LC_{50}$ value of 7.2nM was obtained in MDA 468 cells. However, in populations exposed to 10μM or 30μM DF 129 or DF 203, proliferating colonies were observed: MDA 468 cells revealed a second $LC_{50}$ value > 30μM. In contrast, following exposure periods > 72h (DF 129) or > 96h (DF 203) a more conventional dose response is obtained. Morphological features characteristic of apoptosis were observed as cells, exposed to 10μM and 30μM drug, die.**

| | R |
|---|---|
| CJM 126 | H |
| DF 129 | I |
| DF 203 | $CH_3$ |

**Examination of proteins involved in cellular regulation reveal: decreased p53 expression in MDA 468 cells following 72h treatment with 300nM and 30μM DF 129 as well as reduced expression after exposure to 30μM CJM 126; modulation of bcl-2 expression by benzothiazoles in MCF-7 cell populations. Bcl-2 expression was markedly reduced in cells following 24h treatment with CJM 126, DF 129 and DF 203 (30μM). Expression of bcl-2 appeared unchanged after 72h 30nM DF 203 exposure but a significant decrease in expression was observed in samples treated with 300nM, 3μM and 30μM DF 203. In contrast, enhanced expression of bcl-2 was encountered in LT-10nM and LT-10μM, two MCF-7 variant cell lines exhibiting acquired resistance to CJM 126 ($IC_{50}$ > 50μM) and cross resistance to DF 129 and DF 203 ($IC_{50}$ > 30μM).**

1.3 INVESTIGATIONS INTO THE MECHANISM OF ACTION OF CB30865; A LIPOPHILIC QUINAZOLINE ANALOGUE OF THE ANTIFOLATE ICI 198583.

L.A. Skelton*, M.G. Ormerod, F.D. Mitchell, J. Titley, L. Brunton and A.L. Jackman. CRC Centre for Cancer Therapeutics at The Inst. of Cancer Res., Sutton, Surrey, UK.

CB30865 is a lipophilic analogue of the antifolate ICI 198583 (2-desamino-2-methyl-$N^{10}$-propargyl-5,8-dideazafolic acid) where the glutamate residue of the latter is replaced with an aminomethyl 3-pyridine moiety. It is a potent inhibitor of cell growth *in vitro* (e.g. W1L2 human lymphoblastoid 72 hour $IC_{50}$=3nM). Despite its folate-based structure, CB30865 does not act via inhibition of known pathways of folate metabolism. It is active in the presence of thymidine and other end products of folate metabolism, as well as in TS overproducing cell lines. The biochemical characteristics of CB30865 set it apart from other antitumour agents. For example, a cell line with acquired resistance to CB30865 (W1L2:R865; ~300 fold) was sensitive to all agents tested including antimetabolites, DNA interactive agents, spindle poisons, protein kinase/phosphatase inhibitors, metabolic poisons and various experimental agents. In addition, CB30865 (30nM) induces a gross cell cycle effect in that W1L2 cells arrest simultaneously in all phases of the cell cycle after 20-24 hours exposure. Furthermore, in the NCI *in vitro* screen, CB30865 demonstrated particularly good activity against colon carcinoma and leukaemia cell line panels and was COMPARE negative. In the EORTC *in vitro* screen, CB30865 was found to be inactive against cdc2 kinase and cdc25 phosphatase. Trypan blue exclusion studies revealed that W1L2 cell populations exposed to 30nM CB30865 maintained viability at ~60% after 54 hours but were only 12% viable after 72 hours. The same concentration of CB30865 induced 95% inhibition of colony formation suggesting that those cells viable after 54 hours are in fact destined to die. Intracellular AMP, ADP and ATP levels in W1L2 and W1L2:R865 cells in the presence of CB30865 (30nM) were investigated using HPLC. After 24 hours, the adenylate energy charge (AEC={[ADP]/2 + [ATP]}/[AMP] + [ADP] + [ATP]; an important measure of cellular energy status) was reduced from 0.96 to 0.86 in W1L2 cells (p<0.05) but was unchanged in W1L2:R865 cells. A combination of 2mM KCN/6mM deoxyglucose (DG) reduced the AEC of both W1L2 and W1L2:R865 cells after 24 hours (to 0.87 and 0.93 respectively). However, viability was reduced to a greater extent in W1L2:R865 populations (10 v. 31% control, p<0.05). The relevance of these findings to the mechanism of action of CB30865 is to be evaluated.

1.4 THE *IN VITRO* BIOLOGICAL ACTIVITIES OF SYNTHETIC 18-*O*-METHYL MYCALAMIDE B,10-*epi*-18-*O*-METHYL MYCALAMIDE B AND PEDERIN,

A.Richter[*1], P.Kocienski[2], P.Raubo[2] and D.E. Davies[1]. [1]CRC Medical Oncology Unit, Southampton General Hospital, Tremona Road, Southampton SO16 6YD. [2]Dept. of Chemistry, University of Southampton, SO17 1BJ,

Mycalamides A and B, which were originally isolated from a marine sponge, show close structural similarity to the insect toxin, pederin and exhibit potent cytotoxicity and antitumour activity. Detailed investigation of the clinical potential of these compounds has been hampered because they are available in only minute quantities from natural sources. We now describe the biological activities of 18-*O*-methyl mycalamide B, 10-*epi*-18-*O*-methyl mycalamide and pederin, all prepared by total synthesis. The activities of 18-*O*-methyl mycalamide B and pederin were virtually indistinguishable when evaluated in DNA or protein synthesis assays, and in cytotoxicity assays using human carcinoma cell lines ($IC_{50}$'s 0.2-0.6 nM). In all assays, 10-*epi*-18-*O*-methyl mycalamide B was $10^3$ times less toxic than its diastereoisomer demonstrating that the cytotoxicity of 18-*O*-methyl mycalamide B is inseparable from its ability to inhibit protein synthesis. Short term exposure of squamous carcinoma cells to 18-*O*-methyl mycalamide B or pederin caused an irreversible inhibition of cellular proliferation and induced cellular necrosis. In contrast, the antiproliferative effects of the compounds on human fibroblasts were reversible and there was no evidence of necrosis. Demonstration that 18-*O*-methyl mycalamide B and the synthetically less complex molecule, pederin, show some tumour cell toxicity indicates that this novel class of compounds should, with appropriate modification, be subjected to preclinical evaluation.

### 1.5 NU1025, A POTENT POLY(ADP-RIBOSE) POLYMERASE INHIBITOR, INDUCES APOPTOSIS IN A CELL LINE DEPENDENT MANNER

M.J.Roberts*[1], N.J.Curtin[1], B.W.Durkacz[1], B.T.Golding[2], R.J.Griffin[2], A.G.Hall[1], D.R.Newell[1], S.Srinivasan[2]. [1]Cancer Research Unit, [2]Department of Chemistry, University of Newcastle-upon-Tyne, UK. NE2 4HH

Poly(ADP)ribose polymerase (PARP), is proteolytically cleaved into two fragments of 85kD and 25kD by specific ICE-like proteases during apoptosis. Inhibition of the appropriate ICE-like protease prevents PARP cleavage and also apoptosis, suggesting that PARP cleavage is necessary for apoptosis to occur. The direct cellular effects of a potent and specific PARP inhibitor have been studied. NU1025 inhibits PARP activity with an $IC_{50}$ of 0.4μM in permeabilised L1210 cells. NU1025 was growth inhibitory and cytotoxic against A549 cells (human NSLC), $IC_{50}$ 0.53mM and $LD_{50}$ 0.71mM, respectively. After exposure to 1mM NU1025 for 24hrs, 38 ± 5% of the cells showed morphological characteristics of apoptosis. This level did not increase over time, and at 96hrs 25 ± 6% of cells were apoptotic. Western blotting of A549 cells treated with 1mM NU1025 for 24hrs revealed PARP cleavage. The growth inhibitory $IC_{50}$ values for HT-29 (colon), CCRF-CEM (leukaemia) and MCF-7 (breast) cells were in the range 0.34 - 0.62mM, i.e. similar to A549 cells. However, the degree of apoptosis measured in each cell line following exposure to 1mM NU1025 was markedly different. A549 cells have a relatively high level of apoptosis after 24hrs, while the other cell lines have low levels at early time points which progressively increase. The apoptotic fraction for HT-29, CCRF-CEM and MCF-7 cells at 24hrs was 4 ± 1%, 3 ± 2% and 3 ± 1%, increasing to 17 ± 5%, 36 ± 7% and 12 ± 4% at 96hrs, respectively. FACS analysis of propidium iodide (PI) stained cells revealed no marked phase specific cell cycle arrest induced by 1mM NU1025 in any of the cell lines, although PI binding appeared to increase. In summary, these studies indicate that NU1025 can produce apoptosis in a cell line dependent manner.

### 1.6 AMD473, A NOVEL PLATINUM ANALOGUE, BIPHASICALLY INDUCES P53 AND RESULTS IN INCREASED G2 DELAY AND DELAYED CELL DEATH WHEN COMPARED TO CISPLATIN

J. Holford*[1], B. Murrer[2], L. Kelland[1], [1]CRC Centre for Cancer Therapeutics, Institute of Cancer Research, Sutton, Surrey, [2]Johnson Matthey Technology Centre, Reading.

Our knowledge of the cellular and molecular mechanisms of acquired cisplatin resistance has enabled the identification of the novel platinum-based complex cis-amminedichloro(2-methylpyridine) platinum II (AMD473). This sterically hindered complex displays the ability to circumvent acquired cisplatin resistance in well characterised human ovarian carcinoma (HOC) cell line models, (mean resistance factor = 2 +/-0.4 for JM473 compared to 9.1 +/-3.6 for cisplatin and 7.2 +/-3.6 for carboplatin), where resistance mechanisms are known to include reduced platinum accumulation, elevated glutathione and metallothionein levels, and enhanced DNA repair/tolerance. Also, in our panel of intrinsically resistant HOC cell lines, AMD473 showed only a 30.6 fold increase in $IC_{50}$ value from the most sensitive cell line to the most resistant where with cisplatin it was 117.9 fold.

Alkaline elution studies using HOC cell lines have shown markedly different rates of formation of interstrand DNA cross-links (ISC). With continuous drug exposure, (200μM) ISC formation with CDDP was maximal at 4 hours, where with AMD473, no ISCs could be detected at this time. ISC formation by AMD473 was detectable from 12 hours and was still increasing at 24 hours. In wild type p53 HOC cell lines, the delayed formation of ISCs with AMD473 was accompanied by a biphasic and delayed induction of p53 after treatment with equitoxic concentrations of CDDP and AMD473. AMD473 also exhibited an enhanced G2 cell cycle block which coincided with delayed cell detachment when compared to cells treated with equitoxic concentrations of CDDP.

In conclusion, our *in vitro* data show that AMD473, as well as being able to circumvent many of the cellular resistance mechanisms to platinum agents such as reduced accumulation, glutathione and DNA adduct tolerance/repair, may also be more effective than cisplatin at producing a G2 cell cycle block possibly via altered p53 induction kinetics. This may also explain why AMD473 is less affected by intrinsic resistance mechanisms than cisplatin. Clinical studies of AMD473 are expected to begin in 1997.

This study was funded by the Cancer Research Campaign, U.K.

### 1.7 BIOCHEMISTRY OF POTENT CYTOCHROME $P450_{17\alpha}$ INHIBITORS.

S.Elaine.Barrie[1]*, G.A.Potter[2], F.C.Chan[1], M.Jarman[1]. [1]CRC Centre for Cancer Therapeutics, Institute of Cancer Research, Sutton, Surrey, SM2 5NG. [2]Chiroscience Ltd, Cambridge Science Park, Milton Rd, Cambridge CB4 4WE.

Most cancer of the prostate is initially androgen dependent. Standard treatments, medical or surgical castration, are effective by reducing the level of circulating androgens by ablating the testicular production, but they do not affect the adrenal production of androgens. We have been developing inhibitors of the key enzyme in the androgenic biosynthetic pathway, cytochrome $P450_{17\alpha}$ (17α-hydroxylase/$C_{17,20}$ lyase) as a means of ablating both testicular and adrenal androgen production. Lead compounds of two series were selected for activity against the enzyme from human testes. When tested *in vivo* in mice, abiraterone acetate (17-(3-pyridyl)androsta-5,16-dien-3β-O-acetate), but not CB7645 (2-(4-pyridyl)propan-2-yl 1-adamantanecarboxylate), produced a reduction in circulating androgens and regression of androgen dependent organs. Studies with the murine testicular enzyme showed that CB7645 was a reversible competitive inhibitor with a $K_i$ 10nM. However the $K_m$ for the substrate progesterone was lower at 4.3nM. Abiraterone (17-(3-pyridyl)androsta-5,16-dien-3β-ol), on the other hand, was found to be an "irreversible" inhibitor. The inhibition was slow to occur, and was unaffected by the substrate concentration or by dialysis for 24hr. These studies have been extended to the human enzyme. CB7645 is a reversible competitive inhibitor with a $K_i$ < 1 nM compared with a $K_m$ of 15nM for the substrate pregnenolone. Thus CB7645 is a much more potent inhibitor of the human enzyme than of the murine one. Abiraterone was found to be an "irreversible" inhibitor *in vitro*, its effect being unaffected by 24hr dialysis. Abiraterone and CB7645 are both potent inhibitors of cytochrome $P450_{17\alpha}$ but with different modes of inhibition which may be relevant to their potential clinical use.

### 1.8 XR9051, A POTENT P-GLYCOPROTEIN INHIBITOR, REVERSES MULTIDRUG RESISTANCE *IN VIVO*.

*Mistry, P. Ryder, H. Dale, I. [1]Eccles, S. [2]Plumb, J. [3]Watson, S. Johnson, R. Flatman, K and Bevan, P. Xenova Ltd, Slough, SL1 4EF; [1]ICR, Sutton, SM2 5NG; [2]CRC Dept of Oncology, Glasgow, G61 1BD; [3]Cancer Studies Unit, University of Nottingham, NG7 2UH, UK

Expression of P-glycoprotein (P-gp) is a potential cause of clinical resistance to treatment with many anticancer drugs. XR9051 is a potent and specific inhibitor of P-gp, which reverses drug resistance in several murine and human multidrug resistant (MDR) cell lines. Complete reversal of resistance to a variety of cytotoxic drugs, including vincristine (Vcr), doxorubicin (Dox), and taxol, was observed following 4-6 days coexposure to 0.1-0.5 μM XR9051. In the current study the *in vivo* efficacy of XR9051 was evaluated in murine and human tumour models. The MC26 murine colon carcinoma cell line exhibits intrinsic MDR, which is at least in part, mediated by P-gp. Studies in mice bearing the MC26 tumours demonstrated that coadministration of XR9051, either intraperitoneally or orally (40 mg/kg) significantly (p <0.001) enhanced the antitumour activity of Dox (5 mg/kg, i.v.). XR9051 also significantly potentiated the antitumour activity of Dox in an acquired resistant P388/DXJ murine leukaemia model. Treatment of P388/DXJ tumour bearing mice was performed on days 1, 4 and 7 and the results showed that a significant (p<0.005) increase in median survival time was achieved by Dox (2 mg/kg, i.p.) in conjunction with XR9051 (40 and 60 mg/kg, i.v. and 60 and 80 mg/kg, p.o.) compared to Dox alone. In addition, studies in athymic mice bearing human ovarian (2780AD and CH1/Dox) and small cell lung (H69/LX4) carcinoma xenografts showed that coadministration of XR9051 (i.v. or p.o.) could significantly potentiate the antitumour activity of Vcr, Dox, taxol, epirubicin and etoposide in a dose dependent manner. Moreover, comparison of body weights of animals indicated that the combination schedules of XR9051 plus cytotoxic drug used in these studies were well tolerated. These results demonstrate that XR9051 is a very effective modulator of P-gp mediated MDR and could be beneficial in the treatment of MDR cancers. Phase I clinical studies in healthy volunteers are currently in progress.

### 2.1 PRODUCTION OF MATRIX METALLOPROTEINASE 1 (MMP1) BY BLADDER TUMOUR CELLS FOLLOWING EGF STIMULATION

*J.E.Nutt* and J.Lunec, Cancer Research Unit, Medical School, University of Newcastle, Newcastle upon Tyne, NE2 4HH.*

The matrix metalloproteinases are a family of enzymes which degrade the extracellular matrix and are considered to be important in tumour invasion and metastasis. Levels in bladder cancer have been correlated with tumour grade and invasion[1] and urinary levels of MMP2 have been shown to be elevated in patients with bladder transitional cell carcinoma[2]. Epidermal growth factor (EGF) is found in high concentration in urine and it has been shown that the presence of EGF-receptor (EGF-R) in bladder cancer is a strong independent predictor of tumour stage, progression and poor prognosis[3]. The aim of this work is to study the effect of EGF on MMP1 expression and production in an EGF-R positive human bladder tumour cell line. RT112 cells were grown in serum free medium and treated with EGF (10 or 50ng/ml) for 24 or 48 hours. Following treatment, the conditioned medium was collected and the total MMP1 levels were measured using the Biotrak ELISA assay. MMP1 levels in cell extracts were also measured. Total RNA was extracted from the cells and used for Northern analysis. On Northern analysis MMP2 mRNA remained constant whereas levels of MMP1 mRNA were only detectable in EGF treated samples. Extracellular levels of MMP1 protein in the medium were undetectable in control samples but were increased from 8ng/ml at 24 hours to 24ng/ml at 48 hours of EGF treatment. Levels of MMP1 in all cell extracts remained constant at the lower level of detection (approx. 4ng/ml). These results demonstrate that MMP1 production and extracellular secretion is stimulated by EGF in a bladder tumour cell line. Initial studies have also shown that MMP1 is detectable in urine of patients with bladder tumours and may be important in the progression of EGF-receptor positive tumours and their prognosis.

1 Davies, B. *et al* 1993, Cancer Research **53**, 5365
2 Marguilies, I.M. *et al* 1992, Cancer Epidemiology **1**, 467
3 Mellon, K. *et al* 1995, J. Urol. **153**, 919

### 2.2 THE ROLE OF DESMOGLEIN 2 IN INVASION AND METASTASIS OF BREAST CANCER CELLS.

*E.L.Davies,W.G.Jiang,R.Cochrane,R.E.Mansel. Department of Surgery, Academic Unit,University Hospital of Wales, Heath Park, Cardiff.

Invasion and distant metastasis of human breast cancer cells is a multistep process and of prime importance to the patient. A fundamental step in this process is loss of adhesion of the cancer cells at the primary site.

Desmoglein (Dsg) and Desmocollin (Dsc) are Desmosomal Cadherins which represent a novel subfamily of the Cadherin cell-cell adhesion molecules.They may serve as tumour and metastatic supresser molecules. The aim of this study was to determine the role of Desmoglein 2 (Dsg2) in cell adhesion, invasion and metastasis.

The human breast cancer cell lines MDA MB 231 , MCF 7 and BT 474 were used. The expression of .Dsg2 was demonstrated with Western blotting and Immunocytochemistry.Cell aggregation and invasion was demonstrated with cell-cell aggregation and *in-vitro* Matrigel invasion assays. Cell migration was assessed using Colloidal gold phagokinetic tracking. We compared the breast cancer cells alone and those treated with a Monoclonal antibody (Mab) to Dsg2.

The mean aggregation index (MAI - mean ± standard deviation )was calculated over certain time intervals as shown in the table below.

| TIME (minutes) after antibody addition | 30 | 60 |
|---|---|---|
| MDA cells alone | *0.67±0.14 | *0.78±0.08 |
| MDA + Dsg2 antibody | *0.06±0.32 | *0.13±0.69 |
| BT474 cells alone | *0.69±0.9 | *0.8±0.14 |
| BT474 + Dsg2 antibody | *-0.71±1. | *-0.01±0.81 |
| MCF 7 cells alone | *0.58±0.2 | *0.69±0.13 |
| MCF 7 + Dsg2 antibody | *0.1±0.5 | *0.29±0.46 |

* $p< 0.05$ Wilcoxon Signed Rank Test.

All cells were invasive into the synthetic basement membrane (Matrigel ) and the invasive potential was increased when Dsg2 was blocked with antibody. Cell migration in colloidal gold was also increased in Mab treated cells.

We conclude that Dsg2 is one factor which regulates cell-cell aggregation and *in-vitro* invasion of human breast cancer cells. It is proposed that this adhesion molecule may have an important role in the regulation of invasion and metastasis in patients with breast cancer.

### 2.3 REGULATION OF ENDOTHELIAL CD44 EXPRESSION AND TUMOUR CELL-ENDOTHELIUM ADHESION BY HEPATOCYTE GROWTH FACTOR/SCATTER FACTOR

S. Hiscox and W.G. Jiang. Department of Surgery, University of Wales College of Medicine, Heath Park, Cardiff. CF4 4XN.

Hepatocyte growth factor/scatter factor (HGF/SF) has been implicated in the process of metastasis by its ability to stimulate tumour cell functions such as motility and invasion that are central to the metastatic cascade.

In order for metastasising tumour cells to establish secondary tumours at distant sites, they must enter the circulatory system and thus interact with the endothelial cells lining the circulatory vessels.

CD44 is a cell surface adhesion receptor involved in cell-cell and cell-matrix interactions and has been implicated in a number of biological process including lymphocyte homing and tumour progression.

The aim of this study was to examine the effect of HGF/SF on endothelial CD44 expression and whether this affected tumour cell-endothelium adherence.

Expression of endothelial CD44 following HGF/SF stimulation (40 ng/ml) was assayed using an OPD-based ELISA method together with Western blotting. Tumour cell adherence to HGF/SF-activated endothelial cell monolayers was assessed by co-culturing fluorescently-labelled human colon cancer (HT115) and breast cancer (MCF7) cells with HGF/SF-activated endothelial cell monolayers. Endothelial-bound tumour cells were detected by a fluorescence plate reader.

Following stimulation by HGF/SF, expression of CD44 on endothelial cells was observed to increase in a time dependant manner, with a maximum level response observed following 60 minutes HGF/SF incubation. These results were confirmed by Western blotting studies which revealed up-regulation of a 85-90 kDa protein corresponding to CD44 in response to HGF/SF. The attachment of human colon cancer and breast cancer cells to endothelial monolayers was seen to increase following activation of the endothelial cells by HGF/SF; the increase in endothelial-bound tumour cells observed here could be attributed to the CD44 receptor as inclusion of anti-CD44 antibodies resulted in a reversal of this effect.

We conclude that HGF/SF induces the expression of the cell-cell adhesion molecule, CD44, on the surface of endothelial cells thus facilitating the attachment of tumour cells to the endothelium. These observations may further implicate HGF/SF in the process of metastasis.

### 2.4 GAP JUNCTIONAL COMMUNICATION AND THE TYROSINE PHOSPHORYLATION OF CONNEXIN 43 IN THE INTERACTION BETWEEN BREAST CANCER AND ENDOTHELIAL CELLS.

J Cai, W G Jiang,and R E Mansel. University Department of Surgery, University of Wales College of Medicine, Cardiff, CF4 4XN

Gap junctions play critical roles in maintaining the functional integrity of organs and tissues. Abnormal gap junction communication (GJC) has been associated with the process of carcinogenesis. We investigated the the role of endothelial cell GJC and connexin 43 (Cx43), the main gap junction protein in these cells, during tumour cell extravasation.

Breast cancer cells, MDA MB231, were transferred onto confluent monolayers of human endothelial cells, ECV304. Junctional communication was determined by the ability of Lucifer Yellow-loaded cells to transfer the dye to neighbouring cells. Tumour-endothelial interaction was assessed by 1,1'-dioctadecyl-3,3,3',3'-tetramethylindocarbocyanine perchlorate (DII). Connexin 43 expression and tyrosine phosphorylation were measured by immunoprecipitation and Western blotting.

Co-culturing of ECV304 with MDA MB231 resulted in a rapid and transient loss of communiction competence of ECV304 (shown in the table are number of positive endothelial cells transferred with Lucifer yellow, * vs control by Mann-Whitney U test). The results clearly demonstrated that ECV304 cell communication was profoundly inhibited within 5 min.

| Time (mins) | Positive ECV304 cells | p value* |
|---|---|---|
| 0 | 28.6±1.1 | - |
| 5 | 8.8±0.6 | 0.01 |
| 15 | 14.4±1.5 | 0.01 |
| 30 | 16.8±1.0 | 0.01 |
| 60 | 20.7±1.4 | 0.01 |
| 120 | 28.7±0.5 | 0.7 |

The communication was almost fully restored after 2 h of co-culture with MDA MB231. Addition of tumour cells showed an increase in the tyrosine phosphorylation of CX43 protein as revealed by immunoprecipitation and Western blotting. This pattern was similar to the loss and the recovery of GJC in ECV304 as determined by dye transfer.

We conclude that interaction of tumour cells with endothelial cells effectively inhibits GJC of endothelial cells, which is attributed to the increased tyrosine phosphorylation of connexin 43. This may contribute to the extravasation of tumour cells from the circulation, an essential step in the establishment of metastasis.

2.5 ADHESION OF THREE BREAST CANCER CELL LINES TO HUMAN UMBILICAL VEIN (HUVEC) AND HUMAN BONE MARROW ENDOTHELIAL CELLS (HBMEC). *A O'Callaghan, LC Masek and JW Sweetenham. CRC Wessex Medical Oncology Unit. Southampton General Hospital. SO16 6YD.

Adhesion of malignant cells to endothelium at distant sites is one of the key events in metastatic spread. To further understand this process we have examined the adhesion characteristics of three breast cancer cell lines (MDA-MB-361, MDA-MB-415 & MCF-7) to HUVEC and HBMEC.
The adhesion molecule phenotype of the 3 cell lines was determined by flow cytometry. All cell lines were >95% positive for integrin subunits α3, α5, α6 and β1. Sialyl Lewis X (sLeX) expression was 100%, 30% and 21% respectively for MDA-MB-415, MDA-MB-361 and MCF-7.
Adhesion to endothelial monolayers was examined in a 96-well fluorescence adhesion assay system. Single cell suspensions of breast cancer cells were stained with the fluorescent PKH2 vital membrane dye. $10^4$ cells/well were allowed to adhere to endothelial cells for specified times. Fluorescence intensity before and after washing to remove non-adherent cells was measured using a Cytofluor II fluorescence plate reader and adherence calculated. For antibody blockade, cells or monolayers were preincubated with monoclonal antibodies for 30 minutes, with antibodies remaining throughout the adhesion period.
Only MDA-MB-415 showed a significant increase in adhesion (two-fold) to activated HUVEC above resting HUVEC. Adhesion to HBMEC was similar to activated and non-activated monolayers for all 3 cell lines. Pre-incubation of MDA-MB-415 with anti-sialyl Lewis X or activated HUVEC with anti-E-selectin abrogated the effect of activation of the monolayer. These antibodies had no effect on adhesion of this cell line to HBMEC or on either of the other cell lines to HUVEC or HBMEC. Expression of sLeX predicts for increased adhesion to activated HUVEC, mediated by the E-selectin-sLeX receptor-ligand pair but this mechanism is not used in HBMEC adhesion.
Incubation of the cell lines with antibodies to integrin subunits α5 and β1 caused a substantial reduction in adhesion of MCF-7 (50 & 52%) and MDA-MB-361(35 & 46%) to HBMEC. There was no effect on adhesion of MDA-MB-415 to HBMEC. These antibodies had a detectable but less marked effect on adhesion of all three cell lines to HUVEC.
We suggest that the integrin α5β1, is an important mediator of breast cancer cell adhesion to human bone marrow endothelium.

2.6 COMBRETASTATIN A-4, AN AGENT THAT DISPLAYS POTENT AND SELECTIVE TOXICITY TOWARDS TUMOUR VASCULATURE.

GG Dark[1]*, SA Hill[1], VE Prise[1], GM Tozer[1], GR Pettit[2], DJ Chaplin[1]. [1]Tumour Microcirculation Group, Gray Laboratory, Northwood, Middlesex, HA6 2JR, UK, [2]Dept. of Chemistry, University of Arizona, USA.

Vasculature is critical to both the survival of a solid tumour mass and its continued growth. Indeed tumours cannot grow beyond 1-2 mm in diameter without developing a functional blood supply. Tumour associated endothelium therefore represents a key target for new approaches to cancer therapy. A number of agents can produce vascular mediated cell death in experimental and human tumours, however, only when administered at or close to their maximum tolerated dose (MTD).

Combretastatin A-4 is a constituent of the Zulu medicinal tree *Combretum caffrum*, a species indigenous to South Africa. It is a potent cancer cell growth inhibitor and an effective inhibitor of colchicine binding to tubulin. Combretastatin A-4 and its more soluble sodium (1n) phosphate derivative (prodrug), display concentration-dependent cytotoxicity against a variety of human tumour cell lines, but have enhanced selective toxicity towards tumour associated and proliferating endothelium, when assessed *in vitro* by a viable cell quantitative assay. In contrast, quiescent endothelium is quite resistant to the effects of these agents.

In view of this selective action against tumour associated endothelium, vascular studies were performed in experimental and human breast cancer models *in vivo*. Histology of a variety of human xenografts and murine transplants indicated extensive haemorrhagic necrosis at 24 hours following the systemic administration of a single dose of combretastatin A-4 prodrug at 100 mg•kg$^{-1}$. Despite the profound vascular shutdown in the tumours, there was no evidence of morbidity, nor action against non-tumour tissue. Quantitative assessment of the selective and rapid shutdown in tumour blood perfusion, was demonstrated using a fluorescent vascular perfusion marker, which indicates a reduction in functional vascular volume of 93% at 6 hours following drug administration, with persistence even after 18 hours. There was no corresponding reduction in normal tissue. Following infusion of combretastatin A-4 prodrug into isolated rat tumours, a 3-4 fold increase in vascular resistance, consistent with a similar reduction in blood flow, is evident within 20 minutes. No effect is observed if this solution is infused into the normal hindlimb, indicating the selective nature of these vascular effects.

These studies have identified combretastatin A-4 and its soluble prodrug as agents that can elicit selective effects against proliferating endothelial cells *in vitro*, and induce rapid vascular shutdown within tumours *in vivo*, at doses <10% of the MTD. These selective actions against tumour vasculature, and the prodrug's broad therapeutic window without morbidity, demonstrate the exciting clinical potential of these drugs.

2.7 RETINOID MODULATION OF MELANOMA CELL LYSIS BY LYMPHOKINE ACTIVATED KILLER CELLS AND THE ROLE OF SOLUBLE INTERCELLULAR ADHESION MOLECULE-1
C.L. Alexander, M. Edward and R.M. MacKie, Department of Dermatology, University of Glasgow, Glasgow G12 8QQ.
Intercellular adhesion molecule-1 (ICAM-1, CD54) exists as a soluble form (sICAM-1) in addition to its membrane associated form found on the surface of tumour cells. sICAM-1 has the ability to bind lymphocyte function associated antigen-1 (LFA-1, CD11a/CD18) present on the lymphocyte surface thereby blocking the binding site of LFA-1 for membrane ICAM-1 on tumour cell surfaces. This is thought to be one possible mechanism as to how tumour cells evade immunosurveillance. This study investigates the production of sICAM-1 from the A375 and C8161 human melanoma cell-lines pretreated for 4 days with $10^{-6}$M retinoic acid (RA) and the immunological consequenses of the interaction of sICAM-1 with IL2 activated peripheral blood lymphocytes (PBL). The expression of sICAM-1 within cell-free supernatant, collected over a 4 day period, was determined using an ELISA. Results showed the A375 cells secreted basal ICAM-1 levels of 40.62fg/cell whereas the C8161 cells secreted lower basal levels of 26.03fg sICAM-1/cell. After 4 days pretreatment with $10^{-6}$M RA, sICAM-1 levels were raised to 51.94fg/cell in the A375 cells yet remained unaltered in the C8161 cell-line. The immunomodulatory effects of sICAM-1 were investigated by the addition of sICAM-1 to cytotoxicity assays. IL2 activated/non-activated PBL were added to chromium-51 labelled tumour cells at ratios ranging from 1:1 to 80:1, with the addition of sICAM-1 at the 5:1 and 40:1 ratios. In the A375 cell-line, $10^{-6}$M RA treatment for 4 days did not alter the susceptibility of the tumour cells to lysis by the PBL and addition of sICAM-1 at the 40:1 ratio had little effect on cell lysis. However, at the 5:1 ratio, lysis of the control cells by IL2 activated PBL was reduced from 20.00% to 10.77% and in RA treated cells, from 17.57% to 12.37%. Pretreatment of the C8161 cells with $10^{-6}$M RA induced an increase in lysis at the 20:1-80:1 ratios. Lysis of the C8161 cells on addition of sICAM-1 at the 5:1 ratio was reduced in untreated control cells from 27.11% to 18.19% and was reduced from 22.27% to 3.34% on treatment with RA. At the 40:1 ratio, lysis remained unaltered in control cells but was decreased in RA treated cells from 74.34% to 25.60%.
Collectively, these results provide evidence that sICAM-1 does appear to partially block the interaction between PBL and the melanoma cells although the levels of ICAM-1 required to induce this effect are ten times greater than is secreted from the melanoma cells. Combining these results with additional data from our laboratory, it appears likely that RA treatment of the tumour cells induces changes to several other molecules possibly involved in the tumour/PBL interaction as well as both membrane and soluble ICAM-1 expression.

2.8 ROLE OF bFIBROBLAST GROWTH FACTOR IN PPNET's AND NEUROBLASTOMA *L.Sturla, I.Lewis and S.A.Burchill.Cancer Research Unit, St James University Hospital, LEEDS LS9 7TF.

Peripheral primitive neuroectodermal tumours (pPNETs) and neuroblastoma are common solid tumours of childhood, thought to arise following growth arrest of neural crest cells during development. Nerve growth factor (NGF) and basic fibroblast growth factor (bFGF) have both been implicated in commitment to a neuronal phenotype[1]. We have previously demonstrated NGF and its receptors to be important in the regulation of neuroblastoma cell growth and differentiation[2], but not in pPNETs. In this study we have examined the effect of bFGF on cell growth and differentiation in pPNET (TC-32 and RDES) and neuroblastoma (IMR-32 and SK-N-SH) cells. Proliferation was measured by bromodeoxyuridine incorporation and cell number using a haemocytometer. Cell phenotype was analysed by Western blot and morphology. Effect of bFGF on growth of cell lines in soft agar was also examined. bFGF (5-80ng/ml) decreased incorporation of bromodeoxyuridine in pPNET cells after exposure for 48h. bFGF-induced growth inhibition was accompanied by a decrease in expression of neurofilament protein and loss of neuronal morphology. Cells lost their polar phenotype and became flattened with multiple short processes. This change in phenotype was reversible when bFGF was removed. Both pPNET cell lines grew well in soft agar, colony size and number was reduced in the presence of bFGF (20ng/ml). In contrast, bFGF increased bromodeoxyuridine incorporation and growth of both neuroblastoma cell lines. bFGF had no effect on morphology or neurofilament expression in these cells after exposure for 4 days. Treatment of the IMR-32 but not SK-N-SH cells for 48h induced the cells to pile up, suggesting bFGF may induce a more transformed phenotype in the N-myc amplified IMR-32 cells. SK-N-SH cells did not grow in soft agar, in the absence or presence of bFGF. However, the IMR-32 cells grew in soft agar and treatment with bFGF (20ng/ml) induced a two-fold increase in both colony number and size.
In summary, bFGF decreased proliferation and growth of pPNET cells on plastic and in soft agar. This was accompanied by differentiation into a less neuronal phenotype. In contrast, bFGF increased proliferation and growth of neuroblastoma cells. These results suggest bFGF-induced withdrawal of pPNET cells from the cell cycle may influence the specification of cell phenotype, and may provide a mechanism for decreasing growth of these tumour cells. These effects support the hypothesis pPNET's and neuroblastomas may have derived from different neural cell progenitors.
1. Anderson DJ.(1993). *Ann. Rev. Neurosci.*, **16**:129-158.
2. Burchill SA, Berry PA and Lewis IJ.(1995). *J. Neurol. Sci.*, **133**:3-10.

3.1 **A RANDOMISED PLACEBO CONTROLLED TRIAL OF THE EFFECTS OF MORPHINE-3 AND -6-GLUCURONIDE ON RESPIRATORY CONTROL AND ANALGESIA.**
*Richard T Penson*, Simon P Joel, Krishna Bakhshi, Simon J Clark, Richard M Langford and Maurice L Slevin.* ICRF Department of Medical Oncology and Anaesthetics, St Bartholomew's Hospital, London, UK

Recent reports suggest that morphine-3-glucuronide (M3G) may functionally antagonise the respiratory depressant and analgesic actions of morphine (M) and morphine-6-glucuronide (M6G). To investigate this we have conducted a randomised, double-blind, crossover, placebo controlled study in ten healthy volunteers. The study had six arms; placebo (P), M 10mg/70kg, M6G 3.3mg/70kg, M3G 30.6mg/70kg, M3G with M at the above doses and M3G with M6G at the above doses. Analgesia, assessed using the sub-maximal ischaemic pain model, and toxicity were measured by numerical and visual analogue scales. Respiratory parameters and response to 5% $CO_2$ challenge were assessed by spirometry and mass spectroscopy. Estimation of $PaCO_2$ was by earlobe blood gas analysis.
M6G and M produced significant pain relief compared to P, (pain relief AUC M vs P p<0.001, M6 vs P p=0.033), although pain relief after M6 was less than after M (AUC 4.21 vs 5.63, p=0.009). M3G was not different from P (p=0.26). Pain relief AUC for M and M6G were not significantly altered by M3G (M vs M+M3G, p=0.59, M6 vs M6G+M3G, p=0.28). Dysphoria, sedation and nausea were similar after M and M6G. Significant and similar dysphoria occurred whenever an agonist was administered but was absent with M3G or placebo (*p*<0.003). Ventilation ($lmin^{-1}$ ±SD) following 10mins $CO_2$ was; P 15.6(±4.7), M3G 15.0(±3.8), M 11.2(±4.9), M6G 12.7(±4.1), M+M3G 11.5(±3.0) and M6G+M3G 11.8(±3.9). $CO_2$ challenge caused significantly less respiratory drive in the presence of an agonist (ANOVA *p*=0.003), an effect that was not reversed by M3G (M6GvsM6G+M3G, p=0.35, MvsM+M3G p=0.83). These data were supported by measurements of resting $FeCO_2$ and ear lobe blood gases.
This study is the first placebo controlled trial of the analgesic effects of M6G in man. The data suggest an analgesic potency ratio for M6G:M of around 2:1. This is the first time that the pharmacology of M3G has been examined in isolation in man. M3G appears devoid of significant activity and does not, in these circumstances, appear to antagonise M6G or morphine.

3.2 PHARMACODYNAMIC STUDIES OF THE BIOLOGICAL EFFECT OF THYMIDYLATE SYNTHASE INHIBITORS: FROM LABORATORY TO CLINIC.

D Farrugia*[1], K Danenberg[2], D Cunningham[1], R Metzger[2], P Danenberg[2], HJ Lenz[2], F Mitchell[1], GW Aherne[1], D MacVicar[1], K McCarthy[1], A Norman[1], AL Jackman[1]. [1]Inst. of Cancer Research and Royal Marsden Trust, Surrey. [2]USC, Los Angeles, CA 90033.

*In vitro* studies with the quinazoline based specific thymidylate synthase (TS) inhibitor ZD1694 (Tomudex™) in human tumour cell lines have shown resistance through impaired transport, decreased polyglutamation by down-regulation of folylpolyglutamyl synthetase (FPGS), and increased TS expression (Jackman AL, 1995, Br. J. Cancer, 71: 914). In mice, an inter-strain difference in ZD1694 toxicity is associated with different tissue drug levels. Following 10 mg/kg daily x 5 days, Balb/c mice lose 25% of baseline weight compared to 10% in DBA2 mice. Small bowel drug levels on day 5 were 3.2 ± 1.9 nmol/g in Balb/c and 0.9 ± 0.4 nmol/g in DBA2 mice (p=0.0004, n=3). Clinical studies have reported lower tumour TS expression in responding advanced gastric and colorectal cancer (CRC) (Johnston P, 1995, Cancer Res. 55:1407). This study examines the correlation between tissue drug retention, target enzyme expression, and efficacy/toxicity of Tomudex (3 mg/m$^2$ 3 weekly), in patients (pts) with metastatic CRC. Pts have pre-treatment (Pre-Rx, day -1) and post-treatment (Post-Rx, day 5) CT-guided tumour biopsies during the first Rx course. Same day biopsies of normal rectal or colonic mucosa are also performed. Pre-Rx measurements consist of tissue levels of FPGS and TS mRNA (rt-PCR), and immunohistochemistry (IHC) for TS protein (also in primary tumour) and p53 status. Post-Rx studies include polyyglutamated drug levels (radioimmunoassay; RIA) and TS protein (IHC). Plasma is collected for Tomudex (RIA) and deoxyuridine levels (accomp. abstract). Data from the first 7 patients show an 8 and 4-fold variation in TS mRNA levels in tumour (TS/ß-actin ratios 2-17), and bowel (range 3-11) respectively. Tumour FPGS mRNA varied 35-fold (FPGS/ß-actin ratios 16-560) compared to 14-fold (range 26-368) in bowel. Two thirds of tumours stained positive for p53. Day 5 Tomudex levels ranged from 0.22 to 0.78 nmol/g in tumour, and 0.06 to 0.2 nmol/g in bowel. Day 5 tumour/plasma and bowel/plasma drug ratios were high (90 and 70 respectively) with drug still detected in plasma on day 21 (~ 2 nM). To date, 4 out of 7 pts have shown disease response, 3 of these in the biopsied site. There is an indication of greater bowel toxicity in pts with higher bowel drug levels: grade 0-1 toxicity : 0.10 ± 0.05 nmol/g (n=3); grade 2-4: 0.18 ± 0.03 nmol/g (n=4) (p=0.03). Such correlations may in future improve patient selection and assist dose adjustment. *Supported by the Cancer Res. Campaign and Zeneca Pharmaceuticals.*

3.3 PHASE I STUDY OF THYMITAQ™ IN CHILDREN WITH ADVANCED CANCER.

Estlin EJ[1,2], Pinkerton CR[1], Ablett S[1], Gowing R[1], Lashford L[1], Morland B[1], McDowell H[1], Lewis IJ[1], Kohler J[1], Newell DR[2,4], Taylor GA[2], Boddy AV[2], Johnston A[3], Clendennin N[3], Pearson ADJ[1]. On behalf of the United Kingdom Childrens Cancer Study Group[1], Cancer Research Unit, University of Newcastle upon Tyne[2], Agouron Pharmaceuticals Inc, San Diego, U.S.A.[3] and the Cancer Research Campaign[4].

THYMITAQ™ (AG337) is a non-classical inhibitor of thymidylate synthase, the terminal enzyme involved in the *de-novo* synthesis of thymidylate. Ultimately, the agent may have a role in the therapy of tumours responsive to methotrexate. A multicentre Phase I study of THYMITAQ™, administered as a continuous 5 day intravenous infusion every 28 days via a central line, has commenced for children with advanced cancer. Three patients (pts) were treated at 480mg/m$^2$/24hrs (free base). There were no acute toxicities seen and steady state plasma levels of 5-7μg/ml (mean AUC = 45mg/ml.min; range 41-52) were achieved. Six pts have been treated at the second dose level of 620mg/m$^2$/24hrs (free base), and 3 pts are evaluable for toxicity. No dose limiting toxicities have been observed. In 1 pt, CTC Grade 2 oral mucositis was seen, and in a second pt, Grade 3 mucositis (affecting the upper gastrointestinal tract) and Grade 3 neutropenia and thrombocytopenia were observed. In both patients, all toxicities had resolved by day 12. Pharmacokinetic analyses in five patients at the second dose level have been performed, and steady state plasma levels of 6-10μg/ml (mean AUC = 54mg/ml.min; range 41 - 69) were achieved. At both dose levels, THYMITAQ™ was rapidly cleared from the plasma at the end of the infusion, (elimiation half-life = 1.2 - 2.6 hours). No responses have been observed, although in 2 pts with acute lymphoblastic leukaemia at the first dose level, a 50% reduction in the peripheral blast count was found at the end of the 5 day infusion. The pharmacokinetic results are in keeping with the findings from the adult Phase I study of THYMITAQ™ given as a continuous 5 day infusion. THYMITAQ™ has been sucessfully administered on an outpatient basis, and multicentre pharmacokinetics have been possible . The third dose level of 768 mg/m$^2$/24hrs (free base) has opened, and recruitment to the study continues.

3.4 **The pharmacokinetics of etoposide in combination with cisplatin and carboplatin.**
H.D. Thomas*, D.J. Porter, J.Nobbs, M. Highley, F. Chapman, A.H. Calvert, A.V.Boddy
Cancer Research Unit, University of Newcastle, Newcastle upon Tyne, UK.

Etoposide is frequently combined with either cisplatin or carboplatin in a number of chemotherapy regimens. Studies of combinations of cisplatin or carboplatin with etoposide in patients with testicular teratoma have shown that, although both regimens acheive the same percentage response rate, relapse was more frequent in patients receiving carboplatin (Bajorin, *et al. J. Clin. Oncol.* 11:598-606 (1993)). As single agent treatments for other tumours, carboplatin and cisplatin have been shown to be equally effective at equitoxic doses (Taylor, *et al. J. Clin. Oncol.* 12:2066-2070 (1994)). One explanation for this observation is that either cisplatin or carboplatin interferes with the pharmacokinetics or pharmacodynamics of etoposide (Relling, *et al. Clin. Pharmacol. Ther.* 56:503-511, (1994), Rodman, *et al. J. Clin. Oncol.* 12:2390-2397 (1994)). The objective of this study was to determine the effect of concurrent administration of carboplatin or cisplatin upon the pharmacokinetics of etoposide.

Patients with solid tumours, for whom a combination of etoposide with a platinum drug was appropriate, were studied. All patients had normal liver and renal function, as defined by standard tests. Patients were treated with etoposide 100mg/m$^2$ as a 60 min infusion on 3 consecutive days. On day 2, patients were randomised to receive either carboplatin at a dose based on renal function to achieve an AUC of 5mg/ml.min, or 100mg/m$^2$ cisplatin with saline diuresis. Patients were crossed over to the other platinum drug for the second course. Blood samples were collected throughout days 1-3 for both courses. The plasma concentrations of etoposide were assayed by HPLC. The AUC of etoposide on each day was calculated using the trapezoidal rule.

Ten patients have been studied so far. Of these 7 recieved both combinations, 2 recieved only the cisplatin arm and 1 recieved only the carboplatin arm. The administration of cisplatin did not cause a significant change in the AUC of etoposide, comparing day 2 to day 1. However, the AUC of etoposide on day 2 was significantly increased (median 16%, range -2 to 41%) when carboplatin was co-administered (p=0.007, paired t-test). This effect was reversed on day 3, when the AUC was not different to that on day 1. The order of study of the platinum drugs made no difference to the results. Investigations are continuing into the mechanism of this apparent interaction.

### 3.5 FIBROBLAST GROWTH FACTOR RECEPTOR FOUR EXPRESSION IS INCREASED IN HUMAN COLORECTAL CANCER

A. C. R. Burns *[1], A. C. Gough[1], W. R. Roche[2] and J. N. Primrose[1]. [1]University Surgical Unit and [2]University Pathology, Southampton General Hospital, Southampton. S016 6YD

The Fibroblast Growth Factor (FGF) Family which have been implicated in tumourigenesis act via four tyrosine kinase cell surface receptors (FGFR 1 - 4). Basic FGF (FGF 2) markedly stimulates the growth of human colorectal cancer cell lines, and in solid colorectal tumours has been immunolocalised to carcinoma cells and extracellular matrix components.

The aim of this study was to analyse the expression of FGFR 1 - 4 at the mRNA and protein level in normal and malignant colorectal tissue. Total mRNA was extracted from 22 colorectal cancer / mucosa paired samples previously snap frozen in liquid nitrogen. RT - PCR was performed and the products resolved by polyacrylamide gel electrophoresis and their nature confirmed by cloning and sequencing. To determine the cellular distribution of FGFR, immunohistochemistry was carried out on paraffin embedded colorectal tumours using the streptavidin - biotin complex immunoperoxidase technique with antibodies (Santa Cruz Biotechnology) specific for the carboxytermini of FGFR 1 - 4.

FGFR 1 - 4 was expressed in both normal mucosa and tumour specimens :

| FGFR | Mucosa (n = 22) | Tumour (n = 22) |
|---|---|---|
| 1 | 86 % | 82% |
| 2 | 82 % | 91% |
| 3 | 91% | 95% |
| 4 | 82% | 100% |

The increased expression in tumour compared to the matched mucosa was statistically significant for FGFR 4 ( $p < 0.001$ ), but not for the other FGFR. FGFR 1 - 4 was immunolocalised to the carcinoma cells in addition to normal colonic glands, microvasculature, fibroblasts and smooth muscle.

The apparent up - regulation of FGFR 4 suggests that this receptor may play a role in colorectal tumour development.

### 3.6 BCL-2, PLATELET-DERIVED ENDOTHELIAL CELL GROWTH FACTOR (PD-ECGF) AND TUMOURAL ANGIOGENESIS IN NON-SMALL CELL LUNG CANCER (NSCLC).

KJ O'Byrne*[1], MI Koukourakis[1, 3], A Giatromanolaki[2, 3], KC Gatter[2] and AL Harris[1]. ICRF Medical Oncology Unit[1] and Dept. of Cellular Science[2], Oxford Radcliffe Hospital, U.K. and Dept. of Radiotherapy and Oncology, University Hospital of Iraklion, Greece[3].

Angiogenesis, the formation of new blood vessels, is essential for tumour growth beyond 1 - 2mm in diameter and plays a key role in tumour metastasis. Angiogenesis, assessed by tumour blood vessel counts, is an important prognostic factor in a number of solid tumours including NSCLC. Bcl-2 expression is associated with an improved prognosis and is inversely related to angiogenesis in NSCLC. PD-ECGF overexpression is seen in 25% of NSCLC patients and is positively correlated with angiogenesis.
134 surgical specimens from patients with operable T1,2-N0,1 staged NSCLC were examined for vascular grade (high vs low) immunohistochemically using the anti-CD31 antibody, JC-70. Bcl-2 and PD-ECGF (positive *vs* negative) immunoreactivity was analysed using the monoclonal antibodies clone 100 and P-GF.44C respectively.
Bcl-2 expression correlated with low vascular grade tumours ($p=0.03$) and negative PD-ECGF expression ($p=0.03$). 3/27 (11%) bcl-2 positive cases had high vascular grade tumours *vs* 34/107 (32%) of bcl-2 negative cases. 20/35 (57%) bcl-2 negative cases expressing PD-ECGF were of high vascular grade *vs* 15/72 (20%) bcl-2 and PD-ECGF negative cases ($p<0.0003$). Positive PD-ECGF expression is associated with a poor prognosis ($p=0.01$). This work supports our previous contention that bcl-2 may be a suppressor of tumour angiogenesis and correlates with low PD-ECGF gene expression. The results also suggest the involvement of an oncogene that switches on angiogenesis and confers poor prognosis in cases with absent bcl-2 or PD-ECGF expression.

### 3.7 ANALYSIS OF MICROSATELLITE INSTABILITY IN MULTIPLE TUMORS OF THE LARGE BOWEL.

M.G.Tamassia, G.Rossi, L.Roncucci, M.Pedroni, P.Benatti, C.Di Gregorio, F.Vaccina, R.Fante, M.Ghidoni, A.Percesepe, L.Losi, S.De Pietri, M.Ponz de Leon.
Dipartimenti di Medicina Interna e di Scienze Morfologiche e Medico Legali, Università di Modena.

Synchronous or metachronous are observed in about 4-10% of all patients with colorectal carcinoma. Multiple colorectal neoplasms occur significantly more often in patient with Lynch Syndrome (HNPCC) than in general population. Replication errors (RER), expressed as DNA instability in simple repeated nucleotides of 1-4 base pairs (microsatellites), are frequent in colorectal cancer developing in families with HNPCC, but have been also demonstrated in sporadic colon cancer. We analyses 65 neoplasms from 31 patients with multiple tumors of the large bowel. This sample was selected from the 1,298 registered individuals of a specialized colorectal cancer registry, instituted in our Health Care District. Aim of our study was to assess the frequency of microsatellite instability and its biological significance in multiple tumors. RER were detected by analysis of the microsatellites which seem particularly prone to insertions or deletions of bases in hereditary colorectal tumors. DNA was extracted from normal and cancerous formalin-fixed tissue and amplified by PCR for 35-40 cycles. PCR products were analysed on 6% denaturating polyacrilamide sequencing gel. Tumors with RERs in at least one of five microsatellite loci (D2S123, D17S787, D18S57, BAT40, D9S178) were defined as RER+. We studied 38 synchronous (S) and 27 metachronous (M) tumors. RER phenotype was observed in 13 neoplasms (out of 65, 20%); among these, 11 were S (out of 38, 29%) and 2 M (out of 27, 7%). A fraction of these neoplasms was from HNPCC patients: 9 S and 8 M. RER positivity in HNPCC multiple tumors was 47% (8 out of 17), 77% in S (7 tumors of 9), and 12.5% in M (1 tumor of 8). The percentage of RER+ in S and M sporadic tumors was 13.8% (4 tumors of 29) and 5% (one of 19) respectively. Conclusions: the RER+ phenotype seems infrequent in multiple colorectal malignancies (20% of the cases), at least with the most commonly used primers. However, the expression of the RER+ phenotype seem closely associated with HNPCC; thus, we confirm that the RER assay can be of help in identifying Lynch syndrome in the general population.

### 3.8 GERMLINE MUTATIONS OF hMSH2 AND hMLH1 GENES IN LYNCH SYNDROME.

A.Viel, M.Genuardi, A.Bellacosa, E.Capozzi, M.Anti, M.Ponz de Leon, M.Pedroni, M.G.Tamassia, L.Roncucci, P.Benatti, A.Percesepe, G.Neri.
Istituto di Genetica Medica, Università Cattolica, Roma; Centro Oncologico Aviano, Dipartimento di Medicina Interna, Università di Modena, Italy.

Lynch Syndrome (or HNPCC, for Hereditary Nonpolyposis Colorectal Cancer) is an inherited disease which accounts for 3-5% of all colorectal cancer (CRC) burden. HNPCC diagnosis is based on the so-called "Amsterdam Criteria" requiring the presence, in a family, of three or more relatives with verified CRC in two successive generations, one of whom should be first-degree relative with the other two, and at least one case diagnosed before age 50. The molecular basis of HNPCC has recently been elucidated by identifying the role of defects of DNA mismatch repair genes called hMSH2, hMLH1, hPMS1, hPMS2, from the homologous bacterial genes involved in the maintainance of DNA integrity during cell replication. We have investigated type and frequency of mutations at the hMSH2 and hMLH1 loci in a series of 17 Italian HNPCC families. The methods used for mutation analysis - carried out on DNA extracted from peripheral white blood cells - were: RT-PCR on cDNA for the detection of gross rearrangements, and SSCP (on either cDNA or genomic DNA) followed by direct sequencing, for detecting point mutations. Overall, 9 mutations were identified: 6 were predicted to inactivate mismatch repair function while 3 were considered polymorphisms. The mutated gene was hMSH2 in 2 families and hMLH1 in 4. Four mutations have not been previously reported; these are: 1) a point mutation at the splice donor site in intron 17 of hMLH1, which causes skipping of exon 17 in mRNA; 2) a deletion of exon 3 from hMLH1 mRNA, not associated with sequence changes in the exon itself or in intron-exon borders, 3) a 4-nucleotide deletion in exon 7 of hMSH2, causing premature termination of the reading frame, and 4) a nonsense mutation, occurred at hMLH1 codon 301 as a consequence of a CAG→TAG transition. The other mutations identified were a 3 bp deletion removing a lysine from a stretch of 3 repeated lysines encoded at position 616-618 of hMLH1, and a GTA→GTT transversion occurring at the splice donor site in intron 5, which causes a deletion of exon 5 of hMSH2. Conclusions: the overall mutation frequency at either of the two loci was 35.3% of all investigated families; four of the six mutations detected in our study were not previously described. Gene carrier test, in asymptomatic individuals, is now feasible in the 6 families where a pathogenetic mutation has been identified.

4.1

### THE ACTIVITY OF SN-38 IN P53 WILD-TYPE AND MUTANT COLORECTAL CANCER CELL LINES.

Robert H. te Poele, Maurice L. Slevin, Simon P. Joel. ICRF Department of Medical Oncology, St. Bartholomew's Hospital, London.

SN-38 is the active metabolite of the topoisomerase I inhibitor CPT-11. We have investigated the effect of SN-38 concentration, exposure duration and drug AUC on activity and cell cycle distribution in HT29 (mut p53) and LS174T (wt p53) human colorectal cancer cell lines. Cells were exposed to SN-38 (0-100 ηg/ml) for 1-7 days. Percentage survival was assessed by the MTT assay, and cell cycle distribution, including apoptotic cells, was determined by flow cytometry. In HT29 cells arrest in G2 developed within 24h of exposure, during which G1 was rapidly emptied at concentrations $\geq$ 10 ηg/ml, with accumulation continuing up to 48h. The extent of accumulation increased to a maximum at 20 ηg/ml (87.7 ± 2.6%) and declined at higher concentrations. After 48 h release from G2 and concomitant increase in apoptotic cells (up to 83.2 ± 2.8%) and decrease in cell survival (down to 4.5 ± 1.4%) was also concentration dependent. There was poor correlation between AUC and survival or apoptosis ($R^2$ = 0.45 and 0.50 respectively). For incubations with the same AUC survival was significantly lower for more prolonged exposures to lower concentrations (i.e. for an AUC of 1440 ηg/ml.h survival at 48 h x 30 ηg/ml = 108.5 ± 10.6%, 72 h x 20 ηg/ml = 83.2 ± 11.6%, 144 h x 10 ηg/ml = 43.5 ± 12.4%, p= 0.0122), suggesting a possible schedule effect. In LS174T cells arrest in both G1 and G2 occurred at all concentrations, and in S at concentrations ≥20 ηg/ml, and was associated with increased p53 (determined by flow cytometric analysis using DO7 Ab) in each case. The onset of apoptosis and the decrease in survival developed within 24h at all concentrations. Apoptosis and decrease in survival continued up to 96 h after which the effect reached a plateau. Thereafter, surviving cells (25-50%) showed continued arrest in G1, G2 and S at higher concentrations, again associated with increased levels of p53. These data suggest that increased sensitivity to lower concentrations of SN-38 and cytotoxicity in G1 is associated with expression of wt p53 and that lack of wt p53 results in delayed cytotoxicity. In LS174T cells with wt p53 pulsed delivery may prove more effective by allowing arrested cells to return to cycle, whereas in HT29 cells with mut p53 continued exposure is effective.

4.2

### FORMATION AND REPAIR OF SEQUENCE SPECIFIC TALLIMUSTINE ADDUCTS AT NUCLEOTIDE RESOLUTION IN HUMAN CELLS.

Grimaldi, K.A.*[1], Beccaglia, P.[1,2], D'Incalci, M.[2] and Hartley J.A.[1] [1]CRC Drug DNA Interactions Research Group, Dept. of Oncology, University College London Medical School, London. W1P 8BT [2]Istituto di Ricerche Mario Negri, via Eritrea 32, 20137 Milano, Italy.

Tallimustine is a benzoyl mustard derivative of distamycin A which is cytotoxic and has anti-tumour activity. Following non-covalent binding at AT-rich sequences in the minor groove of DNA it alkylates with high sequence specificty and in plasmid DNA, naked genomic DNA and in treated human cells it has been shown to form adducts preferentially at 5'-TTTTGA and 5'-TTTTGG sequences in a region of the *TOPO IIa* gene. Using single strand ligation PCR the current study investigated damage and repair of DNA, at the nucleotide level, after tallimustine treatment of Li-Fraumeni Syndrome derived cells (LFS-087) containing a homozygous P53 mutation. A 500bp promoter region of the human *N-ras* gene was chosen for study—it contains two 5'-TTTTGA sites on the non-transcribed strand and none on the transcribed strand. After treatment with 2 - 20 µM tallimustine for 2 hours there was no detectable adduct formation on the transcribed strand. On the non-transcribed strand there was only one major adduct at a 5'-TTTTGA site and minor adducts (approx. 6-fold less frequent) were detected at the other 5'-TTTTGA site and at 5'-GTTTGA & 5'-TATTGA sequences. In naked genomic DNA the adduct pattern was essentially the same except that there was some non-specific binding to guanines at high doses which was seen in both strands. Sequencing of the gene in this region confirmed that the sequence in the LFS cells was identical to the published sequence. After incubation in drug free medium for 24 hours no repair of any adduct was detected at all doses tested, in fact the intensity of the individual adducts increased during this period. These studies have therefore confirmed the high sequence specificity of tallimustine in human cells. The different affinity for the two 5'-TTTTGA sites is probably due to sequence context rather than intracellular protein-DNA structural features since the same pattern was observed in cells and naked DNA. No tallimustine lesions were repaired in these P53 mutant LFS cells despite the fact that they were capable of repairing damage caused by cisplatin. The observed increase in adduct intensity may be due to slow formation of the covalent link with DNA.

4.3

### RESIDUAL DNA DAMAGE AS A RAPID PREDICTIVE ASSAY OF RADIOSENSITIVITY.

A.E. Kiltie*[1], C.J. Orton[1], A. Ryan[2], B. Marples[1], C.M.L. West[1].
[1] Dept. Of Experimental Radiation Oncology, [2] University of Manchester Department of Oncology, Paterson Institute for Cancer Research, Christie Hospital NHS Trust, Manchester M20 4BX.

It has been shown that fibroblast clonogenic survival correlates with late normal tissue responses in cancer patients treated with radiotherapy. However, clonogenic assays take too long to have practical application in routine testing of pre-radiotherapy patients. Residual DNA damage assays using pulsed field gel electrophoresis (PFGE) are more rapid, and a correlation with clonogenic survival has been shown to exist over a wide range of $SF_2$ values.

A study was made of the relationship between residual DNA damage and clonogenic measurements of radiosensitivity in fibroblasts from pre-treatment cervix cancer patients and two radiosensitive skin fibroblast cell strains. Residual DNA damage was measured using PFGE after irradiating radiolabelled, plateau-phase cells at 37°C and allowing 24 hours for repair. DNA damage was expressed both in terms of the residual damage slope (60 - 150 Gy) and the fraction of activity released (FAR) following 150 Gy.

There was a 2.2-fold difference in surviving fraction at 2 Gy ($SF_2$) for the vaginal fibroblasts (0.15 to 0.32) compared to a 4.0-fold difference in residual DNA damage slope. When the two radiosensitive cell strains were included, residual damage slope correlated with $\alpha$ (r=0.82, p=0.002), D bar (r=-0.91, p<0.001) and $SF_2$ (p=-0.79, p=0.004), and for the vaginal fibroblasts alone the correlations were $\alpha$: r=0.66, p=0.053, D bar: r=-0.83, p=0.006 and $SF_2$: r=-0.63, p=0.07. When residual damage was expressed as FAR at 150 Gy, then for all cell strains the correlations were $\alpha$: r=0.78, p=0.004, D bar: r=-0.86, p=0.001, and $SF_2$: r=-0.78, p=0.004, and.for the vaginal fibroblast strains alone the correlations were $\alpha$: r=0.60, p=0.088, D bar: r=-0.75, p=0.02, and $SF_2$: r=-0.62, p=0.077.

This study demonstrates a correlation between residual DNA damage and clonogenic survival for fibroblasts from pre-treatment cervix cancer patients suggesting that residual DNA damage as measured using PFGE may be useful as a rapid predictive assay of normal cell intrinsic radiosensitivity.

4.4

### DNA-TARGETED THERAPY: DIFFERENTIAL CYTOTOXICITY OF ALTERNATIVE RADIODOANALOGUES OF IUDR TO HUMAN GLIOMA SPHEROIDS.

Mairs RJ[1], Neshasteh-Riz A[2], Wheldon TE[1,2], Angerson W[3], Stanton P[3], and Owens J[4]. [1]Dept. Of Radiation Oncology, Univ. of Glasgow, Glasgow, G61 1BD, [2]Dept. of Clinical Physics, Western Infirmary, Glasgow, G11 6NT, [3]Dept. of Surgery, Univ.of Glasgow, Royal Infirmary, G31 2ER, [4]Radionuclide Dispensary, Western Infirmary, Glasgow, G11 6NT

Radioiodinated iododeoxyuridine (IUdR) is a novel, cycle-specific agent which has potential for the treatment of residual malignant glioma after surgery. Since only cells in S phase will incorporate IUdR into DNA, a major limitation to this therapy is proliferative heterogeneity of the targeted, tumour cell population. Using a clonogenic assay end point, we have compared the toxicities of three radiodoanalogues of IUdR - [$^{123}$I]IUdR, [$^{125}$I]IUdR and [$^{131}$I]IUdR - to the human glioma cell line, UVW, cultured as monolayers in exponential and plateau phase of growth and as multicellular spheroids. Monolayers treated in exponential growth phase succumbed most readily to [$^{125}$I]IUdR ($D_0$ = 2.36 kBq.ml$^{-1}$), while [$^{123}$I]IUdR and [$^{131}$I]IUdR were less effective eradicators of clonogens ($D_0$ = 9.75 and 17.11 kBq. ml$^{-1}$ respectively). Plateau phase monolayer cultures were marginally more susceptible to treatment with [$^{123}$I]IUdR and [$^{125}$I]IUdR (40% clonogenic survival) than [$^{131}$I]IUdR (60% clonogenic survival). In contrast, colony formation from cells derived from glioma spheroids after incubation with 40 kBq.ml$^{-1}$ of radiopharmaceutical was inhibited to a greater extent by [$^{131}$I]IUdR than by [$^{123}$I]IUdR or [$^{125}$I]IUdR: clonogenic survival values were 17%, 42% and 35% respectively. It is concluded that IUdR conjugates of the Auger electron emitters $^{123}$I and $^{125}$I killed only cells which were in S phase during the period of incubation with radiopharmaceutical, whereas the superior toxicity to clonogenic cells in spheroids of [$^{131}$I]IUdR was due to cross-fire $\beta$-irradiation. [$^{125}$I]IUdR was more toxic than [$^{123}$I]IUdR when compared on the basis of administered activity. However, in terms of number of molecules of radiolabelled drug, [$^{123}$I]IUdR was approximately ten times more effective than [$^{125}$I]IUdR. These findings suggest that [$^{131}$I]IUdR or combinations of [$^{131}$I]IUdR and [$^{123}$I]IUdR may be more effective than [$^{123}$I]IUdR alone for the treatment of residual glioma.

4.5

### TAXOL RESISTANCE IN A HUMAN OVARIAN CARCINOMA CELL LINE OCCURRING VIA A NON MEMBRANE-MEDIATED MECHANISM.

P.M.Rogers*, S.Y.Sharp and L.R.Kelland. CRC Centre for Cancer Therapeutics at The Institute of Cancer Research, Sutton, Surrey, U.K.

Taxol (Paclitaxel) is a novel tubulin-binding antineoplastic agent recommended for combination with Cisplatin in late stage ovarian cancer.
We have previously reported a taxol-resistant variant of the CH1 human ovarian carcinoma cell line and are continuing investigations into its resistance mechanism. Using 96Hr Sulphorhodamine B assays, the CH1Taxol$^R$ was found to be 12-fold resistant to taxol and non-cross-resistant to the closely related molecule Taxotere. A trend towards sensitivity to microtubule depolymerizing agents was also apparent.
The addition of 6$\mu$M verapamil had no effect on the resistance of CH1Taxol$^R$ to taxol and western blotting of membrane extracts revealed no detectable P-glycoprotein, suggesting that intracellular factors, rather than Pgp-mediated drug exclusion are responsible for the increased resistance. The Multidrug Resistance Associated Protein (MRP) was also undetectable. Using [$^3$H]-taxol, uptake of taxol by CH1 and CH1Taxol$^R$ was measured, revealing that at isotoxic concentrations, CH1Taxol$^R$ contained 5.6-fold higher taxol concentration than the parent line, indicating increased tolerance for intracellular taxol. No significant difference (P>0.05) in the native levels of Glutathione was found between CH1 and CH1Taxol$^R$ and no induction of glutathione by exposure to 10nM taxol was detected. The doubling time of the parent and resistant cell line was not significantly different at 28.2hr and 28.7hr respectively. This corresponds with the 24hr exposure at which taxol cytotoxicity reaches its maximum, all the cells having passed through one entire cycle. The chromosome number of CH1Taxol$^R$ was established and compared to that of the parent line, revealing a 3-fold increase in the proportion of cells containing 80+ chromosomes, possibly indicating an increase in the level of polyploidy in the population.

The resistance of other cell lines to taxol has been reported to lie at the level of tubulin, but no difference in the total levels of both $\alpha$- and $\beta$-tubulin was detectable by western blotting. Resistance mediated via differences in specific tubulin isoform expression is a possibility and is currently being investigated.

4.6

### TRANSCRIPTIONAL ACTIVATION OF THE *mdr1b* GENE IN RAT MAMMARY CARCINOMAS THROUGH AN NF-IL-6 LIKE REGULATORY ELEMENT.

F. Zhang, J. Riley, T.W. Gant, MRC Toxicology Unit, University of Leicester, Lancaster road, Leicester. LE1 9HN

Mammary carcinomas can be quickly and reproducibly induced by the administration of N-nitroso-N-methylurea to Virgin female rats. We have demonstrated a specific induction, by up to twenty fold, of *mdr1b* gene expression in these tumours (Zhang *et al*, Lab. Invest 75, 413 (1996)). We investigated the mechanism of increased *mdr1b* gene expression. Southern analysis indicated that a gene amplification had not taken place. Analysis of *mdr1b* gene transcription rate, by an RT-PCR based method, revealed an increase in tumour tissue. We performed gel mobility shift assays on regions of the rat *mdr1b* promoter known to be involved in the control of basal transcription in the liver (Silverman and Hill Mol Car. 13, 50 (1995)), and which showed homology to footprinting regions in the mouse *mdr1b* promoter known to be important in causing elevated transcription of the gene in mouse hepatomas (Song *et al* JBC 270, 25468, (1995)). We detected binding of a mammary tumour specific protein to one of these regions in the rat *mdr1b* gene promoter region. Mutation analysis identified the consensus region as TGnnnATGT. This region is directly adjacent to one recently identified in the rat as important for mdr gene expression in a variety of cell lines (Zhou *et al*, Cell Growth and Diff. 7, 1369 (1996)). We have not yet identified the protein binding to our region but some homology with the NF-IL-6 binding site (TGnnATGT) is apparent. This is consistent with our observation of increased expression of P-glycoprotein, the protein product of the mdr genes, around areas of necrosis and inflammation, which are known to release the cytokine IL-6. Additionally, NF-IL-6 is activated by protein kinase C, and an increase in the expression, and translocation to the plasma membrane, of these enzymes is often seen in multidrug resistant cells.

4.7

### IDENTIFICATION OF DIFFERENTIALLY EXPRESSED SEQUENCES BY DIFFERENTIAL DISPLAY RT-PCR IN A HUMAN GASTRIC CARCINOMA CELL LINE WITH ACQUIRED RESISTANCE TO MITOXANTRONE,

L Hutchinson*[1], C Challen[2], J Lunec[2], J A Hartley[1], [1] CRC Drug-DNA Interactions Research Group, Department of Oncology, University College London Medical School, London, W1P 8PT, [2]Cancer Research Unit, University of Newcastle upon Tyne, NE2 4HH.

The human gastric carcinoma cell line EPG85257P (1) exhibits a high sensitivity (IC50 0.005µM) to the intercalating anthracendione drug mitoxantrone. A highly resistant variant EPG85257RNOV has been established (IC50 70µM) with an acquired resistance factor of 14000. The resistance mechanisms have previously been shown to be unrelated to P-glycoprotein and showed minimal involvement of topoisomerase II. Differential display RT-PCR has been employed to detect changes in gene expression between the EPG85257 cell lines. Several reproducible differentially displayed bands were detected. Further analysis of the genes displayed was achieved by cloning, sequencing and Northern blotting.
One sequence when searched against Genbank matched rac1, a low molecular weight GTP binding protein in the ras gene family (2). This approximately 200bp sequence was used to probe mRNA Northerns and reproducibly showed a 60% reduction of expression in the resistant cell line. In order to obtain further sequence information the Rapid Amplification of Complementary Ends (RACE) technique was applied. Down regulation of this protein in the resistant cell line may be related to facilitation of exocytosis by reducing the actin cytoskeleton. Vesicles have been reported previously in this cell line following treatment with mitoxantrone (1).
A second sequence matched to a transmembrane glycoprotein when searched against Genbank(3). Northern blotting showed it to be overexpressed by 56% in the resistant cell line. The full length clone was obtained and was used as a probe for Northern blotting confirming the identity of the protein. The function of this protein is unknown.

(1) Dietel, M et al Cancer. Res. 50 6100 (1990)
(2) Didsbury,J J. Biol. Chem. 264 16378 (1989)
(3) Shimada, S Cancer Research 54 3831 (1994)

4.8

### INTERFERON INCREASES ΔpH OF HT29 TUMOUR CELLS,

A.S.E. Ojugo[1,2,3], P.M.J. McSheehy[1,2,3], M.O. Leach[2], I.R. Judson[3] and J.R. Griffiths[1], [1]CRC Biomedical Magnetic Resonance Research Group, St. Georges Hospital Medical School, London SW17 0RE and [2]CRC Clinical Magnetic Research Group and [3]CRC Centre for Cancer Therapeutics, Royal Marsden NHS Trust, Sutton, Surrey, SM2 5PT.

A useful aid in assessing a tumour's response to radiation or chemotherapy is pH. McCoy *et al.*, (Brit. J. Can., **72**, 905, 1995), demonstrated that $^{31}$P Magnetic Resonance Spectroscopy (MRS), can be used as a non-invasive method for both extracellular and intracellular pH ($pH_{ext}$ and $pH_{int}$) measurements, whereby the plasma membrane pH gradient (ΔpH) can be determined. Ladoux *et al.*, (FEBS Letts., **234**, 353, 1990), showed that interferon can activate the $Na^+/H^+$ antiport system in isolated human tumour cells thereby inducing cytoplasmic alkalinization. The aim of this study was to determine if interferon affected the $pH_{int}$ and $pH_{ext}$ of human HT29 colon tumours *in vivo*.

Human HT29 cells ($10^6$/ml) were injected s.c into the flank of nu/nu mice (30 g). $^{31}$P MRS was performed when tumours reached 0.5-1.5 g. Each mouse received 50,000 IU of interferon i.p. at 24-30 hr and 2-4 hr before start of MRS experiments. The $^{31}$P $pH_{ext}$ marker, 3-aminopropylphosphonate (3-APP), was injected i.p. (11 mmol/kg) ~ 20 min prior to acquisiton of $^{31}$P spectra. Control mice received saline instead of interferon. Anaesthetized mice were placed in a SISCO 200-330 spectrometer at 4.7T, with the tumour positioned over a double-tuned ($^1$H/$^{31}$P) coil and maintained at 37°C. $pH_{int}$ was estimated from the chemical shift difference between the endogenous inorganic phosphate and $\alpha$-NTP peaks and $pH_{ext}$ from 3-APP and $\alpha$-NTP peaks.

The $pH_{ext}$, $pH_{int}$ and ΔpH values before and after interferon treatment were calculated as 6.84, 6.98, -0.14, and 6.85, 7.27, -0.42 respectively. Paired t tests showed that the negative ΔpH significantly increased ($p < 0.001$) due to a significant increase in $pH_{int}$ ($p < 0.001$). There were no significant changes in $pH_{ext}$ for the interferon-treated mice ($p > 0.5$), nor in the $pH_{int}$, $pH_{ext}$ and ΔpH of the control mice ($p > 0.2$).

The work presented herein shows that interferon significantly increased the negative ΔpH of HT29 tumours via an increase in $pH_{int}$. Seymour *et al.*, (SMR, **11**, 6449, 1992, abstract), demonstrated that interferon increased the retention of 5-fluorouracil (5FU) in HT29 tumour cells. Work on isolated HT29 cells and Lettre ascites cells showed that the greater the negative ΔpH across the tumour cell membrane the greater the uptake of 5FU by the cells (Ojugo *et al.*, AACR, **36**, 242, 1995, abstract). This suggests that interferon could modulate 5FU activity on tumour cells by altering the tumour plasma membrane ΔpH.

4.9 **Cell cycle linked phosphorylation and cellular localisation regulates Id HLH protein function** Richard Deed[1], Eiji Hara [2], Gordon Peters[2], and John Norton[1]. [1]CRC Department of Gene Regulation, Paterson Institute for Cancer Research, Christie Hospital, NHS Trust, Wilmslow Road, M20 9BX UK. [2]Molecular Oncology Lab, ICRF, Lincolns Inn Fields, London WC2A 3PX, UK.

Members of the Id family of HLH transcription factors function as negative regulators of DNA binding basic HLH proteins, and have been implicated in the control of cell growth, differentiation and tumourigenesis by sequestering various target bHLH proteins into an inactive state that is unable to bind DNA. We have identified two mechanisms by which these functions of Id proteins are regulated. Using transiently transfected Cos cells we show that in the absence of a bHLH, "E" protein target, Id3 protein is localised exclusively to the cytoplasm/perinuclear region. Co-transfection with the E protein E47 results in nuclear translocation of the Id3 protein. Id3 that is associated with the E protein displays an extended half life, while the E protein itself is more rapidly turned over.

Using transiently transfected Cos cells and/or bacterially synthesised Id proteins, we show that Id2 and Id3 are phosphorylated at a single site by a complex of cyclins A or E and cdk-2 *in vitro* and *in vivo*. The phosphorylation state of the Id protein regulates its ability to interact with different target bHLH proteins as assessed by *in vitro* bandshift assays and *in vivo* reporter assays. In addition, transfection of phospho-variant forms of Id proteins elicits marked effects on cell cycle progression. These data identify two of the control mechanisms involved in regulating the function of this class of cell cycle regulated molecule, that represent potential therapeutic targets for the future development of novel anti-cancer strategies.

5.1 INTRACELLULAR AND EXTRACELLULAR EXPRESSION OF THE CARBOXYPEPTIDASE G2 ENZYME FOR ACTIVATION OF A MUSTARD PRODRUG IN GENE-DIRECTED ENZYME PRODRUG THERAPY (GDEPT). C.J. Springer*[1], R.A. Spooner[1,2], Y. Light[2], J. Martin[1], S. Stribbling[1], F. Friedlos[1] and R. Marais[2]. [1]CRC Centre for Cancer Therapeutics at the Institute of Cancer Research, 15 Cotswold Road, Sutton, Surrey SM2 5NG, U.K. [2]CRC Centre for Cell & Molecular Biology at the Institute of Cancer Research, 237 Fulham Road, London, SW3 6JB, U.K.

The gene for the bacterial enzyme carboxypeptidase G2 (CPG2) was expressed internally in mammalian cells. Human tumour cell lines A2780, SK-OV-3 (ovarian adenocarcinomas), LS174T and WiDr (colon carcinomas) were engineered to express constitutively either CPG2 or bacterial β-galactosidase. These cell lines were subjected to a GDEPT regime, using the prodrug 4-[(2-chloroethyl) (2-mesyloxyethyl)amino]benzoyl-L-glutamic acid (CMDA). The lines which expressed CPG2 had enhanced sensitivity to CMDA which was 11-95 fold greater than the corresponding control lines. CPG2-expressing cells and control cells were mixed in differing proportions, then treated with prodrug. Total kill occurred when only 4-12% cells expressed CPG2, indicating a substantial bystander effect.

CPG2 was also expressed tethered to the outer surface of mammalian cells. Three positions were mutated to remove inappropriate glycosylation sites. Human tumour cell lines (as above) were engineered for stable expression of CPG2 on their surface and were subjected to GDEPT regimes with the CMDA prodrug. The lines expressing CPG2 were 8-150 times more sensitive to CMDA than non-expressing control lines. In bystander analyses, total cell kill occurred when 2-19% of cells expressed CPG2 on their surface, indicating significant bystander effects.

These results establish both the internally and the externally expressed CPG2 with the CMDA prodrug system as effective combinations for the GDEPT approach.

5.2

**Conversion of non-immunogenic tumour into an efficient antigen presenting system using gene transfer.** R.G. Vile, S. Castleden, N. Hardwick and H. Chong. ICRF Laboratory of Cancer Gene Therapy, Hammersmith Hospital.

Previously, we have demonstrated the generation of anti-tumour immunity when tumours are ablated with suicide genes. Whilst investigating the underlying mechanisms, we observed induction of an intratumoural infiltrate of macrophages, CD4+ and CD8+ T cells in B16HSVtk+ tumours being killed *in vivo* with ganciclovir. In addition, distinct patterns of cytokine expression were detected within the dying tumours using rtPCR which closely resembles that of a Th1 immune response (IL-2, IL-12, TNF-α and IFN-γ), which may reflect the anti tumour immunity in this system. Using electron microscopy, DNA laddering and flow cytometry we have also shown that B16/HSVtk+ cells die predominantly by necrosis, rather than apoptosis, which may contribute to generation of an inflammatory response within the tumour. We are now studying the relationship between the levels of apoptosis induced by cell killing (using suicide genes or conventional chemotherapeutic agents) and the immunity produced following *in vivo* cell killing in several tumour cell types. These data allow us to propose a unifying model for the generation of anti tumour immunity using gene transfer strategies.

To augment these effects *in vivo*, we constructed bicistronic vectors in which two or more genes are co-expressed. In a situation in which HSVtk or cytokine alone was unable to eradicate B16 primary tumours, co-expression of IL-2 or GM-CSF with HSVtk was able to ablate (IL-2) or reduce the primary tumour burden (GM-CSF). However, GM-CSF/HSVtk was clearly the most effective combination in generating systemic protection. Such vectors should be useful for both direct *in vivo* gene delivery using targeted vectors (see Diaz et al) or for generation of tumour cell vaccines (see Chong et al).

We are also investigating other factors which may influence the interaction of the immune system with tumour cells, including the role the Fas/FasL system and of heat shock protein expression during tumour cell killing. Our data demonstrate that the balance which exists between tumour tolerance and rejection *in vivo* can be effectively manipulated in favour of generating an immunostimulatory intra tumoral environment using gene transfer strategies.

5.3 **ADOPTIVE TRANSFER OF DENDRITIC CELLS CAN DECREASE THE TUMORIGENICITY, AND INCREASE THE IMMUNOGENICITY, OF A MOUSE MELANOMA MODEL IN VIVO.** A.A. Melcher, G. Hutchinson[1], I. Hart[1], R.G. Vile. ICRF Laboratory of Cancer Gene Therapy, Hammersmith Hospital; ICRF/Richard Dimbleby Department of Cancer Research, St Thomas' Hospital, London.

Neither vaccination with irradiated cells, nor surgical excision of a primary tumour, confers protection against a subsequent tumour challenge with B16.F1 cells. In contrast, ganciclovir-mediated *in vivo* killing of established B16 tumours expressing the HSVtk gene generates short term protection to rechallenge (upto 60 days) in all animals and long term protection in 30% of mice, possibly due to enhanced tumour antigen release and presentation.

Therefore, to enhance further the immunogenicity conferred by the HSVtk/GCV system, we have prepared dendritic cells (DC) from C57BL/6 mice syngeneic with B16 cells. Cells cultured over 8 days from bone marrow using GM-CSF showed typical dendritic morphology. In addition, FACS analysis showed positive staining for surface molecules critical for antigen presentation, including MHC Class I and II, B7.1 and B7.2 and functional analysis in an allogeneic mixed lymphocyte reaction showed DCs to be significantly more potent at antigen presentation than splenocytes.

To investigate whether the DCs could enhance the immunogenicity of B16 cells killed *in vivo* with HSVtk/GCV, $10^5$ B16tk+ tumours were inoculated subcutaneously either alone or mixed with $10^3$-$10^4$ freshly isolated DC. Before GCV treatment was initiated, in one representative experiment, 8 of 8 animals developed palpable primary tumours in the group with no added DC; in contrast, only 1 of 8 animals developed palpable tumours when DC were present. All palpable tumours resolved with GCV treatment and, as shown previously, typically 30% of animals were protected against rechallenge in the group which did not receive DC. However, 70% of animals in which DC were present during tumour seeding, and subsequent killing, remained tumour free long term (>3 months).

These data suggest that professional antigen presenting cells at the site of a growing tumour can reduce its tumorigenicity and can further increase the immunogenicity of the tumour cells when they are killed *in vivo* with suicide genes. From these data, we are currently investigating protocols by which adoptively transferred DC can be used to elicit protection against tumours *in vivo*.

5.4

**Hybrid LTR Retroviral Vectors to Confer Tissue-Specific Gene Expression in Melanoma Cells.** R.M. Diaz+, I.R. Hart+, & R.G. Vile*.
+ICRF/Richard Dimbleby Department, St Thomas' Hospital, *ICRF Laboratory of Cancer Gene Therapy, Hammersmith Hospital, London.

We are developing tissue specific vectors for the targeted gene therapy of melanoma. Plasmid vectors containing a 200bp enhancer element (-2.0kbp to - 1.8kbp) of the human tyrosinase promoter linked to a 380bp minimal tyrosinase promoter directed reporter gene expression to levels which reached 50% of that of SV40 in MeWo cells (human melanoma), but were essentially silent in non melanoma cells. Therefore, we have generated retroviral vectors with different combinations of small regulatory sequences inserted into the viral LTR.

Replacement of the viral promoter with the tyrosinase promoter led to a loss of tissue specificity. However, in a panel of 5 melanoma and 5 non-melanoma cell lines, replacement of the viral enhancer with the tyrosinase enhancer conferred tissue specificity and high viral titres ($10^5$ pfu/mL). For example, there was a 6 fold increase of GM-CSF production from MeWo cells infected with the enhancer- replacement vector, relative to an enhancer deleted vector; in contrast, only a 1.4 fold increase was observed in HeLa cells. Interestingly, replacement of the viral enhancer and promoter with the tyrosine enhancer and promoter gave no detectable expression, suggesting that this combination is not compatible with a functional viral LTR. Vectors in which the tyrosine enhancer was cloned upstream of the viral enhancer gave levels of GM-CSF approaching 100% of those produced by the wild type LTR vector in melanoma cells; in contrast, in non melanoma cells, production did not exceed 45% of wild type LTR levels, suggesting that the tyrosinase enhancer can repress the activity of the adjacent viral enhancer. Finally tandem repeats of the core enhancer element are being cloned upstream of the SV40 promoter within the LTR to increase levels of gene expression. A bicistronic cassette, in which HSVtk and GM-CSF can be expressed from a single promoter, has now been cloned into tyrosinase enhancer hybrid LTR vectors. These vectors have been packaged into the FLYA18 cell line to yield high titre virus stocks which should be resistant to human complement and which co-express both therapeutic genes in a tissue specific manner.

5.5 TARGETING RETROVIRAL PARTICLES TO MELANOMA CELLS BY INCORPORATION OF α-MSH PEPTIDE INTO THE VIRAL ENVELOPE K. Sunassee*[1], F. Morling[2], F-L. Cosset[3], S.Russell[2], R Vile[1]. [1]Lab. Cancer Gene Therapy, Hammersmith Hospital, Du Cane Road, London; [2]Cambridge Centre for Protein Engineering, Cambridge; [3]Centre de Genetique Moleculaire et Cellulaire, Centre National de la Researche Scientifique UMR106, Universite Claude Bernard Lyon -I, Villeurbanne, Cedex, France

Many melanoma cells over-express the α-Melanocyte Stimulating Hormone Receptor (α-MSH R). To target gene delivery to melanoma, we engineered the α-MSH peptide into the Moloney Leukaemia Virus (Mo-MLV) envelope. The α-MSH peptide was placed at the N-terminus of the env gene (MSH-SU) or α-MSH together with an IEGR Protease Xa cleavage sequence (MSH-IEGR SU), replaced the natural viral RHKR sequence adjacent to the p15E region of the TM domain. Following transfection into Tel.Ceb6 cells, viral supernatants were generated from pooled populations of phleomycin resistant producer cells. Immunoblotting showed that both types of α-MSH-fusion envelope proteins were incorporated into the viral envelope, as effectively as wild-type ecotropic Mo-MLV glycoproteins. MSH-SU virus infected both NIH3T3 fibroblasts and murine melanoma Melan-A cells, as efficiently as Mo-MLV, as measured by β-gal reporter gene expression. However, human melanoma cell lines, MeWo and HMB-2 were not infected. MSH-IEGR virus did not infect any of the cell lines tested, before or after proteolytic cleavage with protease Xa. Both MSH-IEGR SU and MSH-SU virus were able to penetrate MeWo cells by endocytosis and could infect, only when co-internalised with adenovirus, which aided endosomal escape. MSH-SU virus also showed enhanced binding to MeWo cell surface receptors (4°C), compared to that observed for wild-type virus, however, the levels of binding (~2.5% of MSH-SU virus) were very low. Radioiodinated MSH-SU virus was also able to compete with α-MSH peptide, for binding to MeWo cells at 4°C. Although, MSH-SU is able to bind to cell-surface receptors on MeWo cells, and can penetrate the cells via endocytosis, infection can not occur unless the viral particles escape the endosome. Despite the successful incorporation of the α-MSH peptide into the viral SU, its size and/or its binding affinity may be too small to over-ride the binding of the natural ecotropic segment still present in the fusion env SU. This suggests that a novel peptide insert must achieve a critical size to confer novel binding specificity to Mo-MLV particles for targeted gene delivery. Further characterisation of these novel retroviral particles will be described.

5.6 COMPARISON OF THE FUNCTIONAL ACTIVITIES OF FOUR MUTATIONS AFFECTING THE OLIGOMERISATION DOMAIN OF p53. ME Lomax*[1], DM Barnes[2], R Gilchrist[1], SM Picksley[3], RS Camplejohn[1] [1]Richard Dimbleby Dept. Cancer Res., UMDS, St. Thomas' Hosp., London, SE1 7EH, [2]ICRF Clin. Oncol. Unit, Guy's Hosp., London, SE1 9RT, [3]CRC Cell Transf. Group, Dept. Biochem., Univ. Dundee, Dundee, DD1 4HN.

Two functional assays for p53, an apoptotic assay and the yeast based functional assay for the separation of alleles in yeast (FASAY) were employed to determine the functional defect in a Li-Fraumeni like family described by Barnes et al. (Lancet 1992 **340** 259). The results of these assays suggested that this patient had a p53 related defect which was a germline mutation of the p53 gene. Sequencing of the p53 cDNA and genomic DNA revealed there to be a missene mutation at codon 337 resulting in a substitution of cysteine for arginine. This mutation lies in the oligomerisation domain of the p53 protein and the mutated protein retains some tranactivational activity in yeast as demonstrated by the FASAY. This mutation may lead to the formation of p53 dimers, not tetramers which are required for efficient transactivational activity.

Varley et al. (Oncogene 1996 **12** 2437) described a Li-Fraumeni family in which there was a missense mutation in the oligomerisation domain of p53 leading to a substitution of proline for leucine at codon 344. PepPlot analysis suggests this mutation to be an inactivating mutation as it severely disrupts the structure of the oligomerisation domain and results from the FASAY also point to this mutated protein having no transactivational activity.

These two mutated p53 genes, along with two other interesting oligomerisation mutants (349STOP and 337 Arg to His) have been generated in an E. coli expression plasmid by site directed mutagenesis and expressed in E. coli. The resulting proteins were purified and their ability to bind to DNA and mdm2, their efficiency to bind to DNA, their requirement of activation by Pab421 for binding to DNA and how the proteins oligomerise, eg. tetramer, dimer or monomer, was assessed.

In addition, the four mutations were generated in a mammalian cell expression plasmid also by site directed mutagenesis and the plasmid was transfected into Saos-2 cells. The ability of the resulting proteins to transcriptionally transactivate endogenous target genes, such as p21, induce apoptosis and suppress transformation was also assessed.

5.7 **EXOGENOUS WILD-TYPE *p53* INDUCES APOPTOSIS IN Hep3B,** R. R. Mitry* and N.A. Habib, Department of Surgery, Royal Postgraduate Medical School, Hammersmith Hospital, Du Cane Road, London W12 ONN

Hep3B is a human hepatocellular carcinoma (HCC) cell line that does not express endogenous wild-type *p53* (wt-*p53*) due to a major deletion in the *p53* gene.[1] Using the liposomal transfection reagent DOTAP (Boehringer Manheim Ltd., East Sussex, UK), Hep3B cell cultures were transfected with pC53-SN3 plasmid (pCMV-Neo-Bam vector with wt-*p53* cDNA encoded into it), while the control cultures were treated with DOTAP only. 24 hr post-transfection and using Northern Blotting, and PCR/RT-PCR techniques, the cultures were checked for the transfer and expression of exogenous wt-*p53* gene. The PCR/RT-PCR were carried out in the presence of specific primers for wt-*p53* and neomycin resistance marker gene ($neo^r$) encoded by the PC53-SN3 plasmid. The cultures were checked for the presence/absence of apoptosis, using three techniques: propidium iodide (PI) DNA labeling, DNA fragmentation assay, and electron microscopy (EM). The PCR results showed the presence of $neo^r$ marker gene in the cultures transfected with pC53-SN3/DOTAP and not in the control cultures. The Northern Blotting results showed that there was wt-*p53* expression, this was confirmed by the RT-PCR results, which also demonstrated $neo^r$ expression, only in the pC53-SN3/DOTAP transfected cultures. The fluorescence microscopy appearance of the PI-labelled cell nuclei showed the characteristic apoptotic morphology where chromatin condensation and nuclear fragmentation were observed in the pC53-SN3 transfected cultures compared to the control cultures which were negative. The PI-staining technique was also useful in estimating the efficiency of the cultures growth. The control cultures grew ~5-fold more efficiently than the pC53-SN3 transfected cultures. A characteristic apoptotic large DNA fragments ladder pattern was observed in the pC53-SN3 transfected cultures and not in the control cultures. The EM results confirmed that many of the cells in the pC53-SN3 treated cultures were apoptotic, noticeably by the presence of many apoptotic bodies, with many cells presenting the characteristic signs of apoptosis such as chromatin condensation, margination and nuclear fragmentation. Some of the apoptotic bodies were being phagocytosed by neighbouring non-apoptotic cells.

In conclusion, *in vitro* transfection of the HCC cell line Hep3B with wild-type *p53* gene resulted in wt-p53 transfer and expression which induced apoptosis, confirming that wt-*p53* is a tumour suppressor gene for HCC cells which do not express wt-*p53*.[2]

**References**

1. Bressac B. *et al.* (1990) *Proc. Natl. Acad. Sci. USA* **87**, 1973-1977.
2. Puisieux A. *et al.* (1993) *FASEB J.* **17**, 1407-1413.

**Acknowledgement** - We thank Dr. Bert Vogelstein for his gift the pC53-SN3 plasmid.

5.8 **Temperature Sensitive DNA Damage Response of Wild-type p53 Cells Transfected with the Dominant Negative p53 Mutant (codon 143 val to ala)**

Melanie M Morris*[1], A. C. McDonald[1], N. Jones[2] and R. Brown[1]. [1]*CRC Dept. Medical Oncology, CRC Beatson Laboratories, Switchback Rd, Glasgow, G61 1BD.* [2]*Dept. Cellular and Molecular Biology, School of Biological Sciences, Stopford Building, University of Manchester, Manchester.*

The dominant negative p53 mutant, codon 143: valine to alanine, has recently been shown to exhibit a temperature sensitive phenotype when expressed in a p53 null background (Friedlander P. *et al.*, 1996, *Mol Cell Biol*, 16, 4961). Phenotypic effects were demonstrated by increased DNA binding, transactivational activity, and a switch to wild type conformation at the permissive temperature (32°C). We have further examined the effect of temperature on clonogenic cell survival and cell cycle progression following ionising radiation (IR) in wild-type p53 cells transfected with this mutant p53 (A2780 mp53 143).

Incubation of the parental and vector alone controls at 32°C resulted in loss of plating efficiency (PE: 0.2 ± 0.16% at 32°C, 22.3 ± 7.8% at 37°C). In contrast, the mutant transfectants exhibited an increased ability to clone at 32°C (PE: 4.5 ± 1.8% at 32°C, 22.7 ± 10.9% at 37°C). Western blot analysis of the parental A2780 cell line showed that on incubation at 32°C, a dramatic induction of wild type p53 ensued. Clonogenic assessment of A2780 mp53 143 cell survival at 37°C resulted in a 2.3 fold resistance to IR, as compared to the vector alone controls. Notably, incubation at 32°C for up to 8 hours following IR, resulted in reduction of this IR resistance and longer exposures to 32°C resulted in a sensitivity profile similar to the vector alone control. Consistent with this observation, IP analysis showed increased p53 in wild-type or supressor conformation following incubation at 32°C.

Further, we have demonstrated that A2780 mp53 143 lose the ability to arrest in G1 following IR-induced DNA damage at 37°C. Incubation at 32°C however causes the A2780 mp53 143 to regain an IR induced G1 arrest (ratio of total S-phase cells 24 hrs post IR: unirradiated controls = 1.05 ± 0.4 at 37°C and 0.3 ± 0.1 at 32°C), confirming the tempertaure sensitive nature of this mutant.

We have thus demonstrated that in a wild type p53 background, the dominant negative p53 mutant, codon 143 valine to alanine, exhibits a temperature sensitive p53 response. Furthermore, we now demonstrate that this phenotype is functional in terms of the cellular response to IR-induced DNA damage.

5.9 ONCOLYTIC ADENOVIRAL THERAPY FOR RECURRENT p53(-) HEAD AND NECK CANCER

I. Ganly[1], D. Soutar[1], G. Robertson[2], S.B. Kaye[2], A. Balmain[3]. D. Kirn[4]. [1]Canniesburn Hospital, [2]Beatson Oncology Centre, Western Infirmary and [3]CRC Beatson Laboratories, Glasgow. [4]Onyx Pharmaceuticals, 3031 Research Drive, Richmond, California, USA.

Mutation of the p53 tumour suppressor gene is the most common genetic alteration found in human cancers. It is mutated in over 50% of all human cancers and in head and neck cancer the frequency of mutation is 50-80%. We have a p53 dependent oncolytic adenovirus which selectively replicates in and lyses p53(-) cell lines. Cytopathic effect assays were carried out on a range of p53(-) and p53(+) tumour cell lines. Cells growing on culture plates were infected with an E1B deleted adenovirus DL1520 at 1 and 10 pfu/cell and the cell lines monitored for cytolysis. Immunocytochemistry using an anti-hexon protein fluorescent antibody was carried out at 96 hrs post-infection. DL1520 virus produced cytolysis only in p53(-) cell lines. All cell lines showing cytolysis stained positively with anti-hexon protein antibody indicating viral replication was occurring. p53(-) tumour xenografts were then formed in nude mice and the tumours injected directly with virus. 6 weeks post-injection, partial or complete regression of tumours had occurred. Based on these *in vitro* and *in vivo* studies, we are carrying out a Phase I clinical trial involving direct intratumoural injection of E1B(-) adenovirus into p53(-) recurrent head and neck cancer patients.

6.1 c-ERBB-4 EXPRESSION IN PRIMARY OVARIAN CANCER

SP Langdon, BJB Simpson, KG Macleod, EP Miller, GJ Rabiasz, JF Smyth and WR Miller. ICRF Medical Oncology Unit, Western General Hospital, Edinburgh, EH4 2XU

Members of the ErbB (type I ) family of tyrosine kinase receptors are frequently overexpressed in many human cancers and increased expression of both EGF receptor (ErbB-1) and ErbB-2 have been associated with poor survival in ovarian cancer. ErbB-4 has recently been described (Plowman et al, PNAS, 90, 1746, 1993) and like ErbB-3 is activated by heregulin. These receptors are now recognised to be highly interactive and preliminary evidence from ovarian cancer cell line models suggests that heregulin acting via either ErbB-4 or ErbB-3 can activate ErbB-2 and either stimulate or inhibit ovarian cancer cell growth (Xu et al, Proc AACR, 37, 191, 1996). Although these results suggest a possible functional role for ErbB-4 in ovarian cancer, no data has been published on the incidence of ErbB-4 in primary ovarian cancer.

We have investigated the expresssion of ErbB-4 in a series of ovarian tumours using immunohistochemistry, Western blot analysis and reverse-transcription PCR (RT-PCR). Immunohistochemical analysis of 23 malignant ovarian carcinomas using the C18 polyclonal antibody (Santa Cruz Biotech) indicated expression within all sections examined. Expression was found almost exclusively in epithelial cells with only occasional stromal staining. Co-incubation of the sections with the control peptide against which the antibody was raised blocked staining and suggested specificity. Western blot analysis indicated the presence of a band at 180 kDa consistent with ErbB-4 in all specimens examined but the presence of other bands also. To assess RNA expression, RT-PCR was undertaken on RNA extracted from 25 specimens. All specimens demonstrated a signal for γ-actin but under the PCR conditions used only 8 of these demonstrated a signal for ErbB-4. Of these 8 positive samples, 7 were of serous histology compared to 5 of 16 negative specimens indicating a significant association with serous histology ($p = 0.01$) ; all 8 positive samples were advanced stage (III / IV) compared to 7 of 16 negative samples ($p= 0.001$) and the survival of the positive sample group was significantly reduced compared to the negative sample group ($p=0.0013$; log-rank). Although the inconsistency between protein and RNA expression data requires resolution, it is clear that many primary ovarian tumours express ErbB-4 and expression may be increased in advanced disease.

6.2 FUNCTIONALITY OF THE PROGESTERONE RECEPTOR IN OVARIAN CANCER AND ITS REGULATION BY OESTROGEN

SP Langdon, H Gabra, GJ Rabiasz, AA Ritchie, JMS Bartlett, RA Hawkins, JF Smyth and WR Miller. ICRF Medical Oncology Unit, Western General Hospital, Edinburgh EH4 2XU

There is increasing evidence to suggest that the progesterone receptor (PR) has a functional role in the genesis and growth regulation of ovarian cancer. Recently, a mutation in the PR gene has been associated with a 3-fold increased risk of non-familial ovarian cancer (Kieback et al, Proc AACR, 37,1704,1996). We have previously shown that loss of heterozygosity (LOH) at the site of the PR gene (11q22) correlated with low PR content and was associated with poor survival in oestrogen receptor (ER)-positive tumours (Gabra et al, Clin Cancer Res, 1, 945, 1995). In contrast where there was no LOH, ER levels correlated with PR and ER-rich tumours had good survival suggesting that PR may counter growth-stimulatory effects mediated through the ER.

In a series of 74 primary ovarian carcinomas, PR content was significantly associated with ER content ($p < 0.0001$ : Pearson correlation). Such an association suggests the presence of oestrogen regulation in these tumours. The association was different between histological types being strong for endometrioid ($p< 0.0001$) and mucinous carcinomas ( $p< 0.0001$) and absent in serous ($p=0.31$) and clear cell carcinomas ($p=0.86$). The loss of association for serous and clear cell may well be due to structural alteration at the PR locus (Gabra et al, Clin Cancer Res, 1, 945, 1995). Increased expression of PR was associated with improved survival in this group and patients with a tumour PR content > 40 fmol/mg protein had significantly better survival than patients whose tumours contained < 40 fmol/mg protein ($p=0.0007$ ; log-rank test).

To obtain evidence that PR may be functional in ovarian cancer, we examined the effect of the progestin megestrol acetate (MA) against the PR-positive PE04 ovarian carcinoma xenograft. Subcutaneous administration of MA ( 1.5 mg ; 60-day slow release pellet) resulted in significant tumour growth inhibition. Evidence of oestrogen regulation of PR in this model was obtained by the use of endocrine manipulation. PR content of the PE04 xenograft in adult female nude mice was 145 fmol/mg protein and this fell to 7 fmol/mg in ovariectomised mice and was at a level of 2 fmol/mg protein in male mice. Administration of 17 β-oestradiol ($E_2$) via slow-release pellets increased PR content to 745 fmol/mg protein which coincided with growth-inhibition consistent with the view that PR may mediate a growth-inhibitory response. The levels of PR correlated with serum $E_2$ concentrations. All these data point to PR mediating a growth-inhibitory effect and being regulated by oestrogen in ovarian cancer.

6.3 ER EXPRESSION AT RELAPSE ON TAMOXIFEN PREDICTS FOR SECOND-LINE ENDOCRINE RESPONSE IN ADVANCED BREAST CANCER Johnston SRD *, Saccani-Joti G, Ebbs SR, Smith IE, Dowsett M. Academic Department of Biochemistry and The Breast Unit, Royal Marsden Hospital, London, SW3 6JJ; Mayday University Hospital, Croydon.

It is recognised that response to second-line endocrine therapy in advanced breast cancer is more likely in patients who had an objective response to tamoxifen given as first line therapy for metastatic disease. We have previously shown that in primary tumours with acquired tamoxifen resistance ER expression is often maintained at relapse, although the quantitative levels of expression may be reduced (Cancer Res. 55; 3331-38). In this study we wished to determine whether ER expression and phenotype (PgR/pS2 expression) determined by immuno-histochemistry (IHC) in the tamoxifen-resistant tumour at relapse would predict for response to second-line endocrine therapy. Primary tamoxifen had been given to 29 patients with locally advanced or metastatic breast cancer, who at relapse had a biospy from accessible disease prior to second-line endocrine therapy with either aromatase inhibitors (AI), medroxyprogesterone acetate (MPA), or other antioestrogens. There had been a previous objective response to tamoxifen in 20 patients, with stable disease for 6 months in a further 3 patients. At relapse after a median of 28 months (range 9-106), a further endocrine response was seen in 18/23 (78%) previous tamoxifen responders, compared with only 1/6 (17%) who had progressed on tamoxifen (p=0.001). 16 tumours expressed ER/PgR at relapse on tamoxifen, of whom 15 (94%) responded to further endocrine therapy. In contrast, of the 13 tumours which were ER-/PgR- at relapse only 3 responded to further therapy. ER/PgR expression was a better predictor for second-line response to AI than prior tamoxifen response, as all responding tumours expressed ER/PgR compared with none of the non-responders. The 3 ER-/PgR- tumours at relapse on tamoxifen who responded to further therapy all came from patients treated with MPA. In conclusion ER/PgR expression determined by IHC at relapse on tamoxifen may be a strong predictor for second-line endocrine response, especially to aromatase inhibitors, and could be a useful predicitive test for patients who relapse during adjuvant tamoxifen therapy where prior endocrine response is unknown.

6.4 p27KIP1 PROTEIN IN HUMAN BREAST CARCINOMA: IMMUNOHISTOCHEMICAL STUDY REVEALS A POTENTIAL PROGNOSTIC MARKER, E.J Shuter*[1], J.J. Anderson[2], G.G. McIntosh[2], D. McKenna[2], I. Milton[3], M. Steward[3], C.H.W. Horne[2,3], J. Lunec[1], [1]Cancer Research Unit, University of Newcastle Upon Tyne, UK. NE2 4HH, [2]Dept. of Pathology, RVI, and [3]Novocastra Laboratories, Newcastle Upon Tyne, UK. NE1 4LP

Cdk inhibitors act to inhibit cyclin cdk complexes and prevent cell cycle progression. We have produced a monoclonal antibody to a full length recombinant fusion protein which allows an accurate and reproducible determination of the levels of the cdk inhibitor p27KIP1 expression in routinely processed tissue. The specificity of the antibody has been demonstrated by western blotting using lysates of the mink lung epithelial cell line, Mv1Lu after TGF-ß1 induction. In our immunohistochemical study of 202 primary breast carcinomas 74% displayed enhanced expression of p27KIP1 protein. A significant correlation, p=0.0262 using the log-rank test, was detected between p27KIP1 immunostaining and increased time before relapse in a subset of 53 tumours which were negative for cyclin D1 protein expression. This study suggests that in breast cancers which are negative for cyclin D1 expression, the presence of p27KIP1 could indicate a greater time before relapse, and as such p27KIP1 could prove to be a useful prognostic marker in conjunction with cyclin D1 status.

6.5 PROGNOSTIC SIGNIFICANCE OF HER2 AND HER4 HETERODIMERISATION IN CHILDHOOD MEDULLOBLASTOMA R.J.Gilbertson*[1], R.H.Perry[2], A.D.J.Pearson[1] and J Lunec[1], [1]Cancer Research Unit, The Medical School, University of Newcastle upon Tyne, Newcastle upon Tyne NE2 4HH, [2]Department of Neuropathology, Newcastle General Hospital.

The type I tyrosine kinase growth factor receptor (RTK I) family includes the epidermal growth factor receptor and the related proteins HER2, HER3 and HER4. Recent studies of this receptor family have revealed complex signaling interactions involving the production of ligand mediated heterodimers that are synergistic for the transformation of cells in vitro. We have analysed the expression (measured by immunohistochemistry) and prognostic significance of all four family members and their ligand heregulin-$\alpha$ in seventy cases of childhood medulloblastoma (cPNET) at twenty five year follow up. Various patterns of receptor and ligand co-expression were identified. HER2 and HER4 were the most frequently expressed family members with 53% of cases expressing both proteins.

In multivariate survival analysis with clinicopathological disease features no individual receptor or heregulin-$\alpha$ achieved significance. In contrast, when considered together, co-expression of HER2 and HER4 demonstrated independent prognostic significance (p=0.006) along with tumour mitotic percent index (p<0.0001), craniospinal radiotherapy dose (p=0.001), age (p=0.006) and tumour stage (p=0.014).

Inorder to demonstrate heterodimerisation between HER2 and HER4 in this disease we performed immunoprecipitation and Western blotting analysis in frozen sections from patient primary tumours. HER4 and HER3 co-immunoprecipitated with HER2 confirming the heterodimerisation of these receptors in medulloblastoma.

Finally, we have also analysed expression of the AP-2 transcription factor implicated in the positive regulation of HER2 and HER3 gene transcription in malignant cells. AP-2 expression was significantly related to HER2, HER3 and HER4 expression (p=0.034, p=0.024 and p<0.0001). AP-2 has not previously been implicated in the control of HER4 transcription suggesting that the possible regulation of HER4 transcription by AP-2 should be explored.

6.6 URINARY TISSUE FACTOR LEVELS IN CONTROLS AND PATIENTS WITH CANCER. BA Lwaleed*[1], M Chisholm[1] and JL Francis[2]. [1]Dept. of Haematology, Southampton University, Southampton, U.K. and [2]Hemostasis and Thrombosis Research Unit, Florida Hospital, FL 32701, U.S.A.

Activation of blood coagulation is a common complication of cancer in man and experimental animals[1]. The causes of such activation may be multifactorial, but increased production of tissue factor (TF) the primary trigger of blood coagulation[2], by the host mononuclear cells may be involved. TF is not only produced by human monocytes (mTF) or even tumour cells, but is also found in urine (uTF) where measurements might be of clinical useful[3]. Using a highly standardized one stage kinetic chromogenic assay developed by our group[3], we measured uTF level in controls [healthy volunteers (n=57) and patients with renal stones and a normal ESR (n=30)] and in patients with benign and malignant diseases of the bladder (n=75), prostate (n=106), breast (n=94) and large bowel (n=62). Each benign disease group was divided into inflammatory and non-inflammatory categories. Controls showed no significant difference with the benign groups except in breast and large bowel disease (P<0.05, P<0.05 respectively). However, a significant difference was observed between the controls and benign groups when compared with patient with malignant disease (P<0.001). All malignant groups demonstrated a significant difference when compared with the relevant non-inflammatory disease (P<0.05), but not with the inflammatory benign disease. Cancer patients showed uTF activity above the upper quartile range of the normal control group for bladder (74.4%), prostate (68%), breast (77.3%) and large bowel disease (73%). In conclusion uTF measurements can distinguish patients with malignancy from normal controls and benign non-inflammatory disease, but not inflammatory disease. uTF may therefore have a role in screening patients for certain malignant diseases.

References:
1) Francis JL. *Med Lab Sci.* 1989; 46: 331.
2) Nemerson Y. *Semin Haemost.* 1992; 29: 170.
3) Lwaleed BA, Chisholm M, Francis JL. *Br J Haem.* 1996; 93: 18.

### 6.7 QUANTITATIVE FISH: CHROMOSOME 9 LOSS A MARKER OF RECURRENCE IN TCC BLADDER?

JMS Bartlett[1], ADWatters[1], JJ Going[2], KM Grigor[3] & TG Cooke[1] Glasgow Univ. Dept. [1]Surgery, [2]Path, G31 2ER.. [3]Univ. Dept. Path. Edin, EH8 9AG.

Estimation of chromosomal copy number, by pericentromeric repetitive DNA sequences in interphase nuclei, provides valuable information in a number of diseases including cancer. This study describes quantitative determination of chromosome 9 copy number by fluorescence in situ hybridisation (FISH) in transitional cell carcinoma of the urinary bladder (TCC). Fixed tissue was used without disaggregation.

Observer and tissue related variability were evaluated for two measures, mean signal/nuclear ratio (MSNR) and percent monosomic cell population (MCP). Intra and inter observer variation for MSNR was consistently lower than for MCP (Intra observer 6.35% vs. 29.73%, inter observer 4.00% vs. 15.02% respectively). In 98% of carcinomas analysed, consistency within multiple tumour areas suggested that multiclonality/tumour heterogeneity were uncommon at this locus; mean intra tumoural variation for MSNR was 6.1% and for MCP was 16.19%. Neither stage nor grade increased the variability of either scoring measure.

Investigation of TCC patients classified by recurrence and/or progression demonstrated that loss of chromosome 9 occurs early. 86 TCC from 33 patients were analysed for loss at this locus using MCP & MSNR. Normal ranges were defined for a control tissue (epidermis) and values for MSNR below 1.51-2.10 denoted monosomy. 22 TCC were detected with monosomic scores. Of 71 TCC, where multiple areas were analysed, 69 (97%) demonstrated the same chromosome 9 copy number in all areas (2-6); 2 tumours showed heterogeneity at this locus. Loss of chromosome 9 persisted in all subsequent TCC from patients who had lost this marker in their primary TCC. 30% (6/20) of patients with subsequent carcinoma recurrence demonstrated loss of chromosome 9 in their primary and all subsequent TCC analysed. Patients (n = 11) with non-recurrent TCC did not show loss of chromosome 9 in their TCC (p = 0.047).

This study describes rapid quantitation of chromosomal copy number by (FISH). Strict quality control demonstrates that this technique is reproducible in a clinical environment and can be used to detect genetic changes relevant to patient outcome. Loss of chromosome 9 indentified patients at high risk of recurrence.

### 6.9 Allelotype of Barrett's adenocarcinoma ; a search for novel tumour suppressor genes

K Dolan, J Garde[2], A Swift[2], J Gosney[1], S Khan, SJ Walker, R Sutton, JK Field[2].
Departments of Surgery and [1] Pathology, Royal Liverpool University Hospital, Liverpool, L7 8XP.
[2]Molecular Genetics and Oncology Group, Clinical Dental Sciences,University of Liverpool, Liverpool L69 3BX.

Loss of function of tumour suppressor genes have been implicated in the development of dysplasia and carcinoma in patients with ulcerative colitis. We have performed loss of heterozygosity (LOH) studies on 22 cases of adenocarcinoma occurring in Barrett's oesophagus, in order to determine the role of tumour suppressor genes in the Barrett's metaplasia--dysplasia--carcinoma progression.

Tissue was obtained from the tumour and from normal gastric mucosa (control), and snapped frozen before microdissection. The genome of each tumour was studied using 110 microsatellite markers, covering all nonacrocentric autosomal chromosome arms.

The sites most frequently demonstrating LOH were found on **3p** (56%), **5q** (50%), **9p** (50%), **11p** (44%), **13q** (50%), **17p** (81%), **17q** (94%) and **18q** (69%). At least seven microsatellite markers were used for each of these chromosomal arms. LOH on 3p, 9p, 13q, 17p and 18q occurred mainly at the sites of the VHL, MTS 1, Rb, p53 and DCC tumour suppressor genes respectively.

The greatest area of LOH on 5q occurred at a site distant to the APC tumour suppressor gene, indicating the possibility of another tumour suppressor gene on 5q. Similarly, the majority of LOH on 11p did not involve the WT1 tumour suppressor gene. The refined areas on 5q and 11p are thus putative sites of novel tumour suppressor genes involved in the development of Barrett's adenocarcinoma. It is of note that the greatest degree of allelic imbalance was found on 17q (94%), with 68% of tumours displaying LOH at 17q11.2-q12. We propose that this is the site of the main tumour suppressor gene associated with the development of adenocarcinoma in Barrett's oesophagus.

Fractional allele loss (FAL) was calculated for each tumour, and the median FAL was 0.22. FAL was not significantly related to the stage of the tumour nor prognosis. However, tumours displaying LOH at the site of the MTS 1 tumour suppressor gene had a significantly higher FAL than those retaining heterozygosity, and all of these tumours with LOH at this site demonstrated microsatellite alterations with other microsatellite markers. LOH at the site of the MTS 1 tumour suppressor gene may be associated with widespread genomic instability.

### 6.8 WAF1 EXPRESSION IN TRANSITIONAL CELL CARCINOMA OF THE BLADDER,

K.L.Braithwaite*[1], J.K.Mellon[2], D.E.Neal[2], C.H.W.Horne[3], C.Challen[1], R.Abdel-Fattah[1], T.R.L.Griffiths[2], K.M.Wood[3] and J.Lunec[1], 1Cancer Research Unit, [2]Department of Surgery, The Medical School, University of Newcastle Upon Tyne, Newcastle, NE2 4HH, [3]Department of Pathology, Royal Victoria Infirmary, Newcastle Upon Tyne, NE2 4LP.

The expression of WAF1, a downstream mediator of p53-dependent cell cycle arrest, was studied using immunohistochemistry in 173 cases of assorted stage and grade of transitional cell carcinoma of the bladder. The WAF1 specific staining of tumour cells was nuclear and often heterogeneous throughout the section. The staining of a higher percentage of cells in the papillary regions of low stage tumours was particularly striking. Low stage tumours contained a significantly higher percentage of WAF1 positive nuclei than muscle-invasive tumours (p=0.0002, Mann-Whitney test). High grade G3 tumours had a significantly lower WAF1 score than lower grade G1 or G2 tumours (p=0.0056 and p=<0.0001, respectively, Mann-Whitney test). A significant inverse relationship was found between the levels of protein detected by immunohistochemistry for WAF1 and p53 ($p=1.46 \times 10^{-5}$, regression analysis) and WAF1 and the cell proliferation marker Ki67 (p=0.024, regression analysis). No significant association was found with WAF1 positivity and tumour progression, progression-free interval or tumour recurrence rate in superficial disease. In the patient group with invasive (T2-T4) tumours, a high percentage of WAF1 positive cells was associated with better survival by log-rank test, which reached significance (p=0.034) when the classification into high and low staining groups used a cut-off of 60%.